# Celecoxib and octreotide synergistically ameliorate portal hypertension via inhibition of angiogenesis in cirrhotic rats

**DOI:** 10.1007/s10456-016-9522-9

**Published:** 2016-07-05

**Authors:** Jin-Hang Gao, Shi-Lei Wen, Shi Feng, Wen-Juan Yang, Yao-Yao Lu, Huan Tong, Rui Liu, Shi-Hang Tang, Zhi-Yin Huang, Ying-Mei Tang, Jin-Hui Yang, Hui-Qi Xie, Cheng-Wei Tang

**Affiliations:** 1 Division of Peptides Related with Human Diseases, State Key Laboratory of Biotherapy, West China Hospital, Sichuan University, Chengdu, People’s Republic of China; 2 Department of Gastroenterology, West China Hospital, Sichuan University, Chengdu, 610041 People’s Republic of China; 3 Department of Human Anatomy, Academy of Preclinical and Forensic Medicine, West China Medicine College, Sichuan University, Chengdu, People’s Republic of China; 4 Laboratory of Stem Cell and Tissue Engineering, State Key Laboratory of Biotherapy and Regenerative Medicine Research Center, West China Hospital, Sichuan University, Chengdu, People’s Republic of China; 5 Department of Gastroenterology, The Second Affiliated Hospital of Kunming Medical University, Kunming, People’s Republic of China

**Keywords:** Angiogenesis, Celecoxib, Octreotide, Portal hypertension, Hepatic arterioportal fistulas, Liver cirrhosis

## Abstract

Abnormal angiogenesis is critical for portal hypertension in cirrhosis. Except for etiological treatment, no efficient medication or regime has been explored to treat the early stage of cirrhosis when angiogenesis is initiated or overwhelming. In this study, we explored an anti-angiogenesis effort through non-cytotoxic drugs octreotide and celecoxib to treat early stage of cirrhotic portal hypertension in an animal model. Peritoneal injection of thioacetamide (TAA) was employed to induce liver cirrhosis in rats. A combination treatment of celecoxib and octreotide was found to relieve liver fibrosis, portal venous pressure, micro-hepatic arterioportal fistulas, intrahepatic and splanchnic angiogenesis. Celecoxib and octreotide exerted their anti-angiogenesis effect via an axis of cyclooxygenase-2/prostaglandin E2/EP-2/somatostatin receptor-2, which consequently down-regulated phosphorylation of extracellular signal-regulated kinase (p-ERK)–hypoxia-inducible factor-1α (HIF-1α)–vascular endothelial growth factor (VEGF) integrated signaling pathways. In conclusions, combination of celecoxib and octreotide synergistically ameliorated liver fibrosis and portal hypertension of the cirrhotic rats induced by TAA via the inhibition of intrahepatic and extrahepatic angiogenesis. The potential mechanisms behind the regimen may due to the inactivation of p-ERK–HIF-1α–VEGF signaling pathway.

**Electronic supplementary material:**

The online version of this article (doi:10.1007/s10456-016-9522-9) contains supplementary material, which is available to authorized users.

## Introduction

Portal hypertension represents the leading cause of mortality and liver transplantation in patients with cirrhosis. Increased intrahepatic vascular resistance and portal venous blood flow are supposed as the major pathological progresses in development of portal hypertension [1]. As a clinical treatment, transjugular intrahepatic portosystemic shunt is the best way to decrease the structural intrahepatic resistance. To reduce the splanchnic blood flow, nonselective β-blocker and vasoactive drugs are widely used. However, all of those modalities are the last resort for the advanced or decompensated cirrhotic patients [2]. Likewise, new medication or regime should be explored to treat the early stage of cirrhosis when angiogenesis is initiated [3–5].

Cyclooxygenase-2 (COX-2), a rate-limiting enzyme involved in the conversion of arachidonic acid to prostaglandins and thromboxanes, is up-regulated in cirrhotic liver [6]. It has been demonstrated that COX-2 inhibitors could inhibit angiogenesis in hepatocellular carcinoma (HCC), breast cancer and other solid tumors [7–11]. Moreover, our recent studies revealed that inhibition of COX-2 through a selective inhibitor celecoxib ameliorates portal hypertension in an animal model [12, 13]. Moreover, such effect is related to its inhibition of intrahepatic angiogenesis and epithelial-to-mesenchymal transition of hepatocytes [12, 13]. Somatostatin (SST) and its analogue octreotide are widely used for the management of bleeding from gastroesophageal varices in patients with cirrhosis [14]. It is notable that octreotide could inhibit angiogenesis in HCC and in the early stage of portal hypertension induced by partial portal vein ligation [15, 16]. Our previous studies have shown that the combination of COX-2 inhibitor and somatostatin analogue synergistically enhanced the anti-angiogenesis effect in HCC [16, 17]. In this study, we aimed to investigate the effects of celecoxib in combination with octreotide on portal hypertension, and intrahepatic and extrahepatic angiogenesis. The potential mechanisms behind the regimen were also investigated through in vivo and in vitro experiments.

## Materials and methods

### Animals and grouping

Sprague–Dawley rats weighing 200–250 g were obtained from Experimental Animal Center of Sichuan University (Chengdu, China). The animal procedures were approved by the Animal Use and Care Committee of Sichuan University and were conducted according to regulations set by Sichuan University. Peritoneal injection (i.p.) of thioacetamide (TAA, Sigma-Aldrich, St. Louis, MO, USA) was used to induce liver cirrhosis (200 mg/kg every 3 days for 16 weeks). 60 rats were randomized into control, TAA and TAA+ combination groups with 20 rats in each group. Control group received normal saline (1 mL i.p., every 3 days); TAA group received TAA; TAA+ combination group received TAA plus celecoxib (gastric gavage, 20 mg/kg/day, Pfizer, New York, NY, USA) and octreotide (intramuscular injection, 50 μg/kg/day, Novartis, Basel, Switzerland). Celecoxib and octreotide were given from the initiation of TAA administration.

### Other methods

Methods for hemodynamic measurements, histopathological evaluation, vascular casting, quantitative real-time PCR (qRT-PCR), immunohistochemistry staining, Western blot, enzyme-linked immunosorbent assay (ELISA), ink-gelatin-dextran perfusion for hepatic arterioportal fistulas (hAPF), Cell culture and treatments, immunocytofluorescence staining, wound-healing assay, tube formation assay and chromatin immunoprecipitation assay (ChIP) are described in the supporting information.

### Statistical analysis

All data were expressed as mean ± SD and were analyzed by SPSS 19.0 software (SPSS, Chicago, IL, USA). For multi-group comparison, one-way ANOVA followed by SNK multiple comparison test was implemented. Natural Log transformation was utilized to transfer non-normal distribution variables into normal distribution variables. A value of *p* < 0.05 was considered significant.

## Results

### Amelioration of liver fibrosis by the combination treatment

**Fig. 1:**
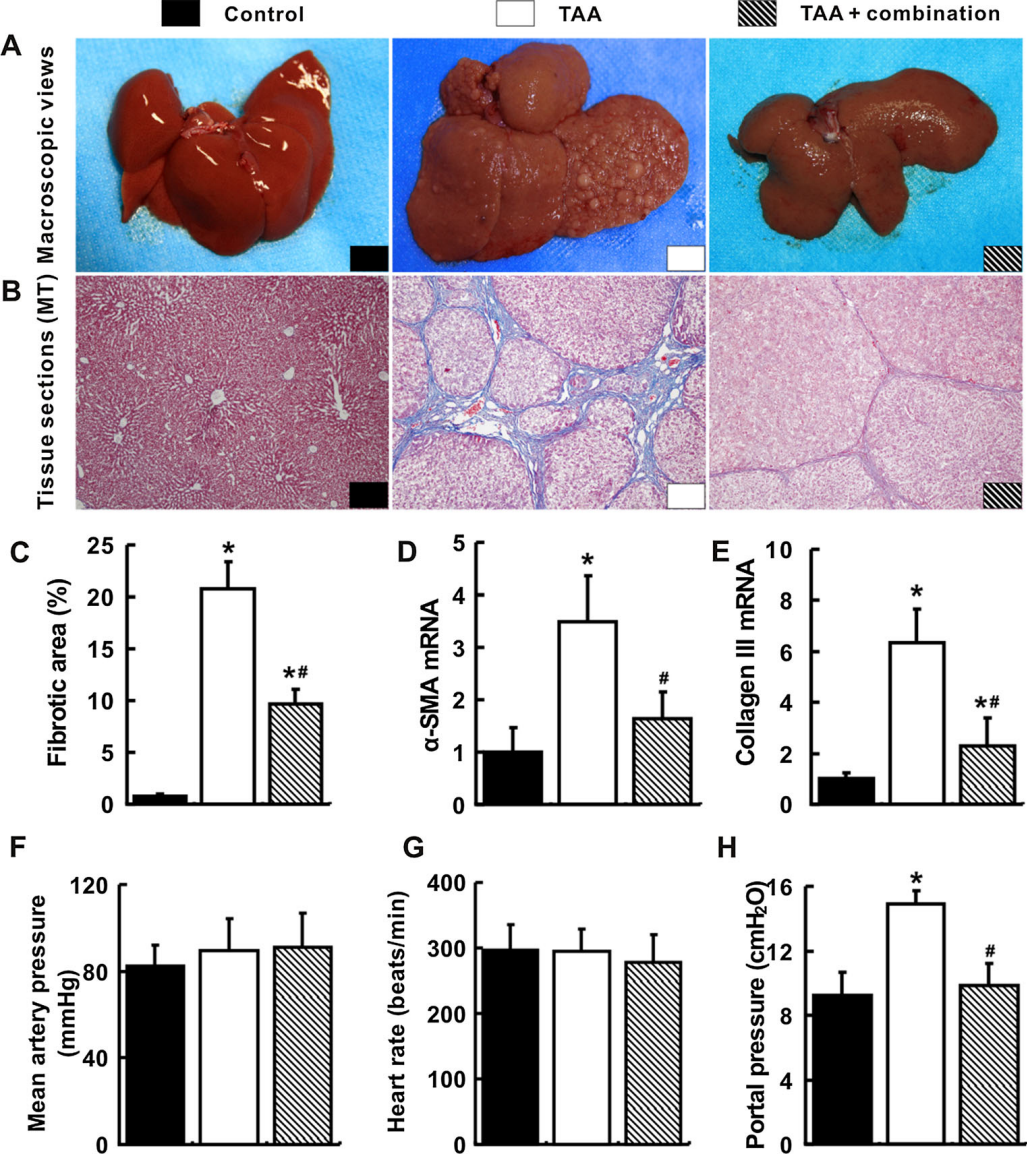
Attenuation of liver cirrhosis and portal hypertension by the combination treatment. Typical cirrhotic appearance with extensive nodular formation (**a**) and fibrotic septa (**b**) was presented in livers of the TAA group. These hepatic nodules and fibrotic septa (Masson’s trichrome staining, ×100 magnifications) were almost not observed in the TAA+ combination group. The fibrotic areas (**c**) and hepatic α-SMA (**d**) and collagen III (**e**) mRNA quantified by quantitative real-time PCR (qRT-PCR) also decreased in the TAA+ combination group. Mean arterial pressure (**f**) and heart rate (**g**) were comparable in three groups. Portal pressure in the TAA group was the highest among three groups (H). *p < 0.05 versus control group; ^#^ *p* < 0.05 versus TAA group

Compared with the control group, liver tissue in the TAA group showed a typical cirrhotic appearance with extensive nodular and continuous fibrotic septa (Fig. 1a, b). The quantitation of fibrotic area of liver tissues in the TAA group increased significantly by 27-folds compared with that of the control group (*p* < 0.05, Fig. 1c). In support of this, hepatic mRNA for collagen III and alpha smooth muscle actin (α-SMA) in the TAA group were 6.4 and 3.5 folds higher, respectively, than those in control group (*p* < 0.05, Fig. 1d, e). Impressively, the hepatic nodules and fibrotic septa reduced greatly in the TAA+ combination group (Fig. 1a, b). The quantitation of fibrotic area and hepatic mRNA for collagen III and α-SMA also decreased significantly in the TAA+ combination group compared with the TAA group (*p* < 0.05, Fig. 1c–e).

### Reduction of portal hypertension by the combination treatment

There were no significant differences of the mean arterial pressure and heart rate among three groups (*p* > 0.05, Fig. 1f, g). A significant increase of portal venous pressure in the TAA group by 60.9 % was noticed compared with that in the control group. However, the portal venous pressure decreased substantially in the TAA+ combination group (*p* < 0.05, Fig. 1h).

### Inhibition of hepatic angiogenesis by the combination treatment

**Fig. 2:**
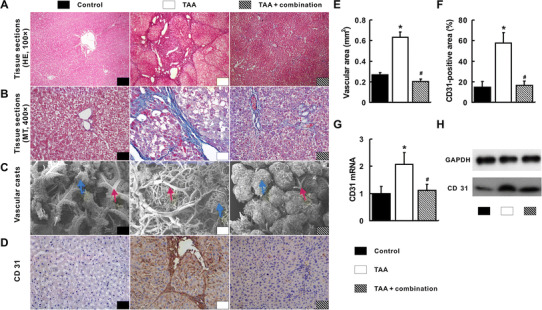
Reduction of hepatic angiogenesis by the combination treatment. Enlargement of hepatic vasculature in the TAA group (**a**) was shown. Angiogenesis was accompanied with fibrosis (**b**). A much more irregular, tortuous portal vein in the TAA group and relative normal portal vein in other groups were visualized by hepatic vascular casts (**c**). Immunohistochemistry (IHC) for CD31 displayed a much more positive staining in the TAA group than other groups (**d**). The hepatic vascular areas (**e**), CD31-postive areas (**f**), CD31 mRNA and protein quantified by qRT-PCR (**g**) and Western blot (**h**) decreased in the TAA+ combination group. *Red arrow* indicates portal vein; *blue arrow* indicates sinusoidal. *p < 0.05 versus control group; ^#^ *p* < 0.05 versus TAA group. (Color figure online)

Liver angiogenesis in the context of fibrosis was illustrated through conjunction of HE and MT staining. As shown, neovascularization was distributed predominantly along the fibrotic septa (Fig. 2a, b). Hepatic vascular areas in the TAA group increased significantly compared with those in the control group (*p* < 0.05, Fig. 2e). These results were further supported by liver vascular casts of portal veins, which showed much more irregular, tortuous portal veins in the livers of the TAA group (Fig. 2c). Furthermore, substantially increased vessel density, mRNA and protein expression of CD31 in the TAA group were found compared with those in the control group (*p* < 0.05, Fig. 2d, f–h). Differently, vascular areas reduced significantly in the TAA+ combination group. The intrahepatic vascular casts of portal vein were less irregular compared with the TAA group (Fig. 2a–c, e). Consistently, the reduced vessel density, mRNA and protein expression of CD31 in the TAA+ combination group were also observed compared with the TAA group (*p* < 0.05, Fig. 2d, f–h).

### Reduction of micro-hepatic arterioportal fistulas (micro-hAPF) by the combination treatment

Ink perfusion assay detected micro-hAPF in TAA and TAA+ combination groups, but not in the control group (Supporting Fig. S1A). Compared with that in the TAA group, the average number of micro-hAPF per liver in the TAA+ combination group reduced significantly (*p* < 0.05, Supporting Fig. S1B).

### Suppression of splanchnic angiogenesis by the combination treatment

**Fig. 3:**
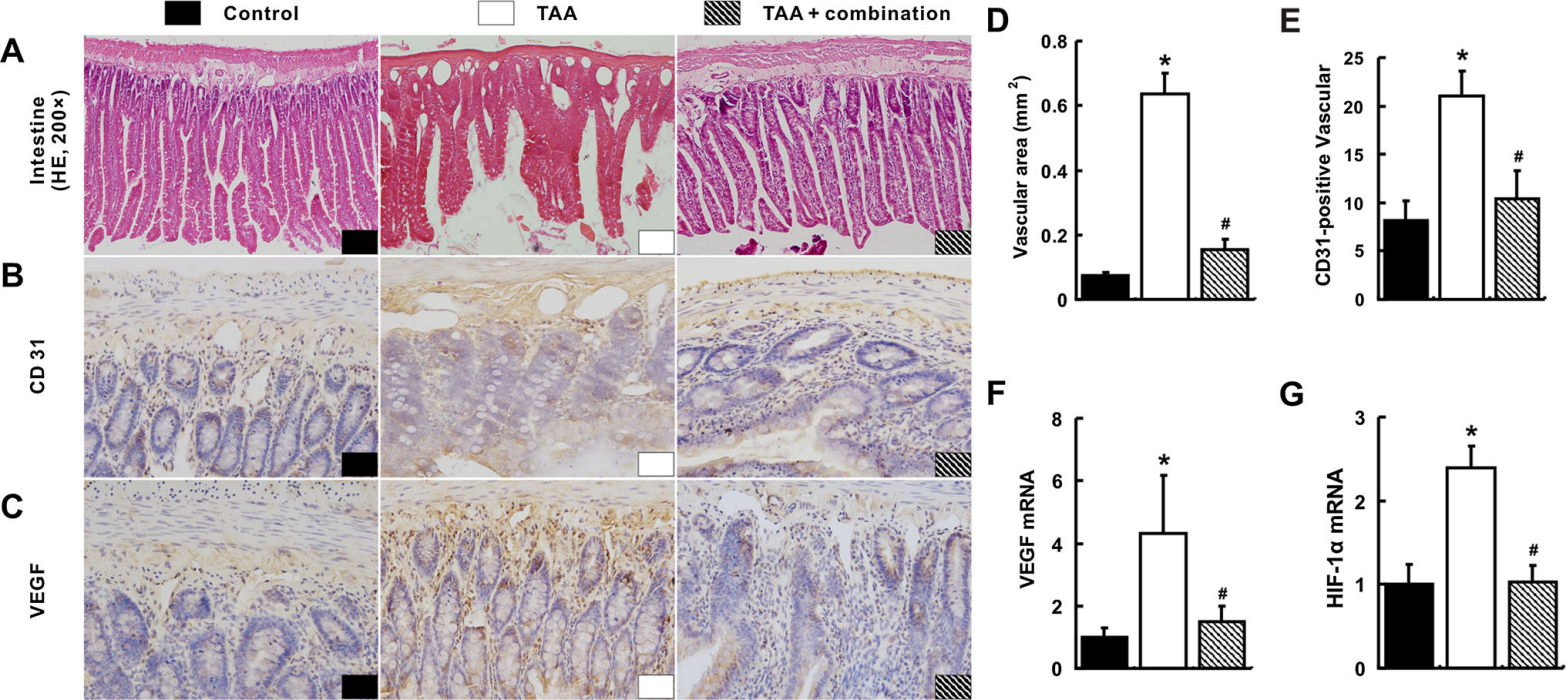
Inhibition of intestinal angiogenesis with the combination treatment. The increased intestinal angiogenesis in the TAA group was visualized by HE (**a**), IHC for CD31 (**b**) and IHC for VEGF (**c**). Intestinal vascular areas (**d**), number of intestinal CD31-postive vessels per fields (**e**), intestinal VEGF (**f**) and HIF-1α (**g**) mRNA quantified by qRT-PCR in the TAA group were the highest among three groups. *p < 0.05 versus control group; ^#^ *p* < 0.05 versus TAA group

Compared with that in the control group, the intestinal vascular area, CD-31 positive vascular and vascular endothelial growth factor (VEGF) expression increased greatly in the TAA group (*p* < 0.05, Fig. 3a–f). The mRNA expression of hepatic hypoxia-inducible factor-1α (HIF-1α) in the TAA group was the highest among three groups (*p* < 0.05, Fig. 3g). However, substantial reduction of those parameters was observed in the TAA+ combination group (Fig. 2a–g). Similar images and data were also obtained from the stomach (Supporting Fig. S2). There was no significant difference of intestinal extracellular signal-regulated kinase mRNA among three groups (*p* > 0.05, Supporting Fig. S3A). However, the activation of intestinal c-Fos and c-Myc was much higher in TAA model than that in the TAA+ combination group (*p* < 0.05, Supporting Fig. S3B, C).

### Inhibition of the signal pathways related to intrahepatic angiogenesis by the combination treatment

**Fig. 4:**
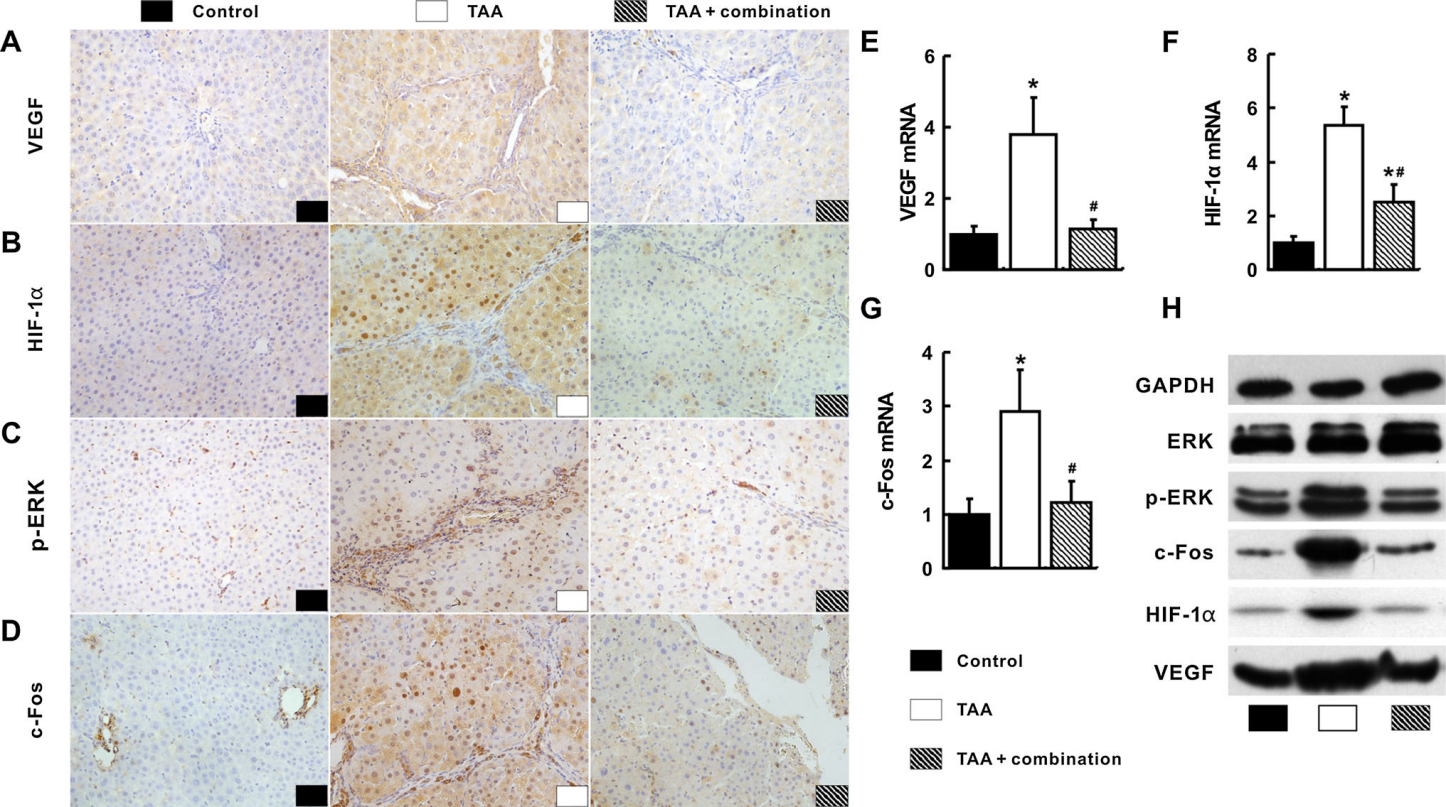
Suppression of the integrated signal pathways with the combination treatment in the liver. Most positive staining of VEGF (**a**), HIF-1α (**b**), p-ERK (**c**) and c-Fos (**d**) visualized by IHC were observed in the TAA group. Consistently, VEGF (**e**), HIF-1α (**f**) and c-Fos (g) mRNA and protein (**h**) and p-ERK protein (**h**) in the TAA group were the highest among three groups. *p < 0.05 versus control group; ^#^ *p* < 0.05 versus TAA group

At protein levels, VEGF, HIF-1α, phosphorylated ERK (p-ERK) and c-Fos in the TAA group were increased obviously compared with that of the control group (Fig. 4a–d). In support of these findings, the mRNA and protein levels of VEGF, HIF-1α and c-Fos and p-ERK protein of the TAA group were the highest among three groups, while they were significantly decreased in the TAA+ combination group (*p* < 0.05, Fig. 4e–h). Consistently, other angiogenic factors such as platelet-derived growth factor BB chain (PDGF-BB) and fibroblast growth factor-2 (FGF-2), which were increased in the TAA group, were also suppressed when treated with celecoxib and octreotide (*p* < 0.05, Supporting Fig. S4A, B). The mRNA levels of ERK and c-Myc did not showed any difference among three groups (*p* > 0.05, Supporting Fig. S4C, D).

### Suppression of the axis of COX-2/PGE2/EP-2/p-ERK/VEGF by celecoxib

The protein and mRNA levels of intrahepatic COX-2, as well as serum concentration of prostaglandin E2 (PGE2) in the TAA group, were found to significantly increase compared with those in the control group. However, the suppression of COX-2 and PGE2 was revealed in the TAA+ combination group (*p* < 0.05, Supporting Fig. S5A-D).

**Fig. 5:**
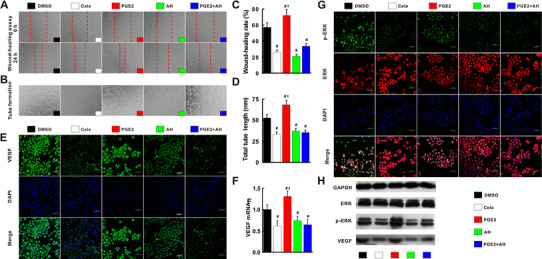
Celecoxib inhibited angiogenesis via inactivation of COX-2/PGE2/EP-2–p-ERK–VEGF signal pathway. Compared with DMSO-treated HUVEC cells, migration rate (**a**, **c**) and tube formation (**b**, **d**) was suppressed by cele, AH and PGE2 + AH, but exacerbated by PGE2 (**a**, **c**). VEGF protein (**e**, **h**) and mRNA (**f**) and p-ERK (**g**, **h**) expression measured by immunofluorescence, and qRT-PCR and Western blot were restored by treatment with cele, AH and PGE2 + AH, but enhanced by treatment with PGE2 compared with DMSO-treated cells. AH, PGE2 receptor EP-2 inhibitor AH6809; Cele, COX-2 inhibitor celecoxib. ^#^ *p* < 0.05 versus DMSO; ^‡^ *p* < 0.05 versus Cele

Compared with dimethyl sulfoxide (DMSO)-treated human umbilical vein endothelial cell line (HUVEC), the migration rate, tube length, p-ERK and VEGF expression in celecoxib-treated cells decreased significantly. As the product of COX-2, PGE2 enhanced those parameters when compared with celecoxib- and DMSO-treated HUVEC cells. Since EP-2 is a key receptor of PGE2, it was not surprising that the migration rate, tube length, p-ERK and VEGF expression were suppressed by EP-2 inhibitor AH6809 when compared with DMSO- and PGE2-treated cells. These inhibitions were also observed in cells co-treated with PGE2 and AH6809 (Fig. 5a–h).

### Inhibition of the cascade of SST/SSTR-2/p-ERK/VEGF by octreotide

The mRNA levels of SSTR-1, SSTR-2 and SSTR-5 but not SSTR-3 or SSTR-4 were significantly up-regulated in the livers of the TAA group when compared with those in the control group (Supporting Fig. S6A). SSTR-2 and SSTR-5 could be detected in HUVEC (Supporting Fig. S6B).

**Fig. 6:**
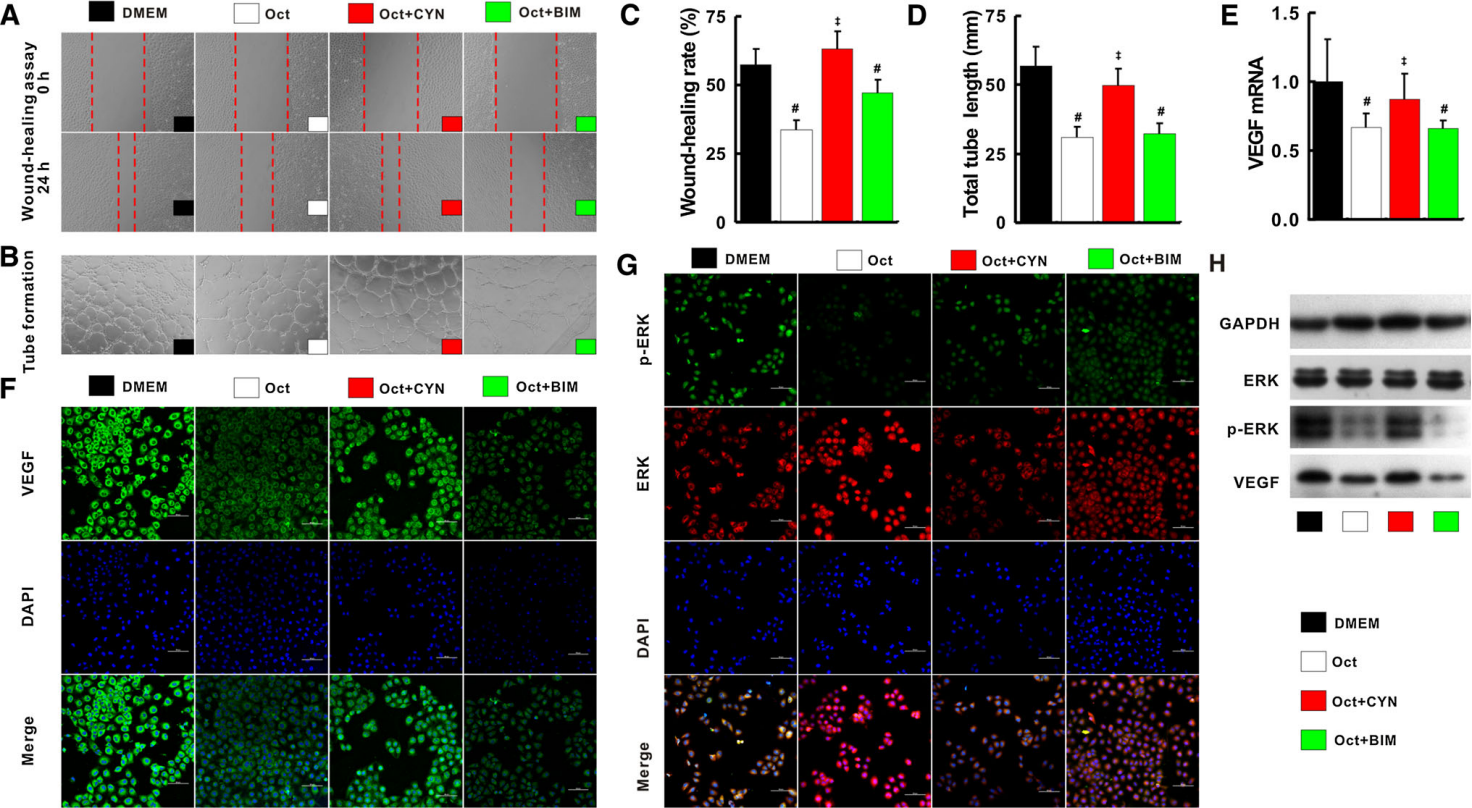
Octreotide suppressed angiogenesis through SSTR-2–p-ERK–VEGF signaling pathway. Compared with DMEM-treated cells, the migration rate (**a**, **c**) and tube length (**b**, **d**) was decreased by Oct and Oct + BIM, but unchanged by Oct + CYN. Similarly, VEGF mRNA (**e**) and protein (**h**), p-ERK (**g**, **h**) level measured by qRT-PCR and Western blot was abolished by Oct and Oct + BIM, but uninfluenced by Oct + CYN compared with DMEM-treated HUVEC cells. Oct, octreotide; CYN, SSTR-2 antagonist CYN154806; BIM, SSTR-5 antagonist BIM23056. ^#^ *p* < 0.05 versus DMEM; ^‡^ *p* < 0.05 versus Oct

Compared with Dulbecco’s modified Eagle’s medium (DMEM), the migration rate, tube formation, p-ERK and VEGF expression were greatly inhibited by octreotide. The inhibitory effects of octreotide could be subtracted with the addition of SSTR-2 antagonist (CYN154806), but not SSTR-5 antagonist (BIM23056) (Fig. 6a–h).

### Synergistical inhibition of p-ERK–HIF-1α–VEGF by celecoxib and octreotide in vitro

**Fig. 7:**
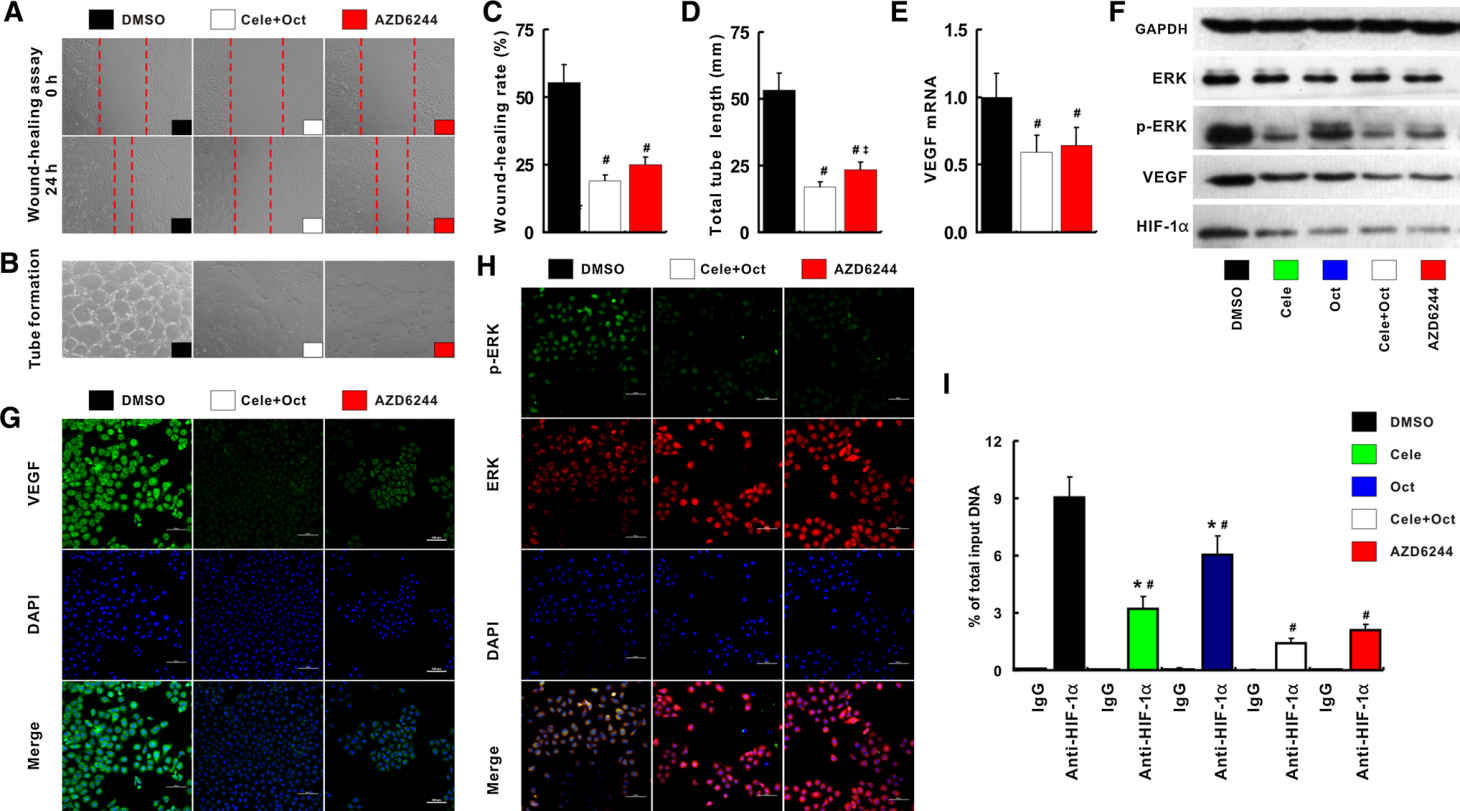
Celecoxib and octreotide synergistically inhibited angiogenesis via p-ERK–HIF-1α–VEGF. Cele + Oct or AZD could significantly reduce the migration rate (**a**, **c**) and tube formation (**b**, **d**) compared with DMSO-treated HUVEC cells. The VEGF mRNA (**e**) and protein (**f**, **g**) p-ERK (**f**, **h**), HIF-1α protein (**h**) determined by immunofluorescence, and qRT-PCR and Western blot were also abolished by Cele, Oct, Cele + Oct or AZD. ChIP assay was to determine HIF-1α binding to VEGF promoter region (**i**). AZD, MEK inhibitor AZD6244. ^#^ *p* < 0.05 versus DMSO. *p < 0.05 versus Cele + Oct

The migration rate, tube length, p-ERK, HIF-1α and VEGF expression reduced significantly with the combination treatment of celecoxib and octreotide compared with the treatments with DMSO, celecoxib or octreotide alone. Interestingly, compared with DMSO-treated cells, these parameters also reduced significantly with blockade of MAPK-ERK signal pathway with AZD6244 (Fig. 7a–h).

### Prevention of HIF-1α binding to VEGF promoter with the combination treatment

In IgG negative control, no HIF-1α binding to VEGF promoter in all of treatments could be measured (Fig. 7i). Conversely, using anti-HIF-1α antibody, we detected recruitment of HIF-1α in the VEGF promoter by the cells treated by DMSO (9.06 ± 1.07 % of the input), indicating activation of the pathway and activation of VEGF transcription. However, the recruitments of the transcription factor were significantly reduced after the treatments in the following levels: celecoxib (3.21 ± 0.63 %), octreotide (6.06 ± 0.97 %), celecoxib + octreotide (1.41 ± 0.26 %) and AZD6244 (2.08 ± 0.32) (*p* < 0.05, Fig. 7i).

## Discussion

In the cirrhotic liver, proliferated cells (hepatic stellate cells and fibroblasts) require sufficient blood flow and nutrition to sustain their proliferation status. Consequently, angiogenesis is enhanced in response to a variety of proangiogenic stimuli [3]. Accumulated data reveal that intrahepatic angiogenesis is driving force of portal hypertension due to a tortuous vascular network of varying diameter and flow pattern which was organized into micronodules and macronodules, enhancing intrahepatic vascular resistance [1, 4]. Moreover, increased vascular bed size of the portal venous system mediated by angiogenesis significantly contributes to increased portal venous blood flow [18]. Our study observed not only intrahepatic but also extrahepatic angiogenesis in the process of portal hypertension in a cirrhotic rat model induced by TAA. Moreover, it was speculated that extensive intrahepatic angiogenesis might lead to the formation of micro-hAPF, an intrahepatic communication between the hepatic artery and the portal venous system. Micro-hAPF would exacerbate portal hypertension by introducing a tremendous arterial blood flow into the intrahepatic portal venous system. To our knowledge, it was the first time to visualize the supposed formation of micro-hAPF in this study. Such confirmation suggests the crucial role of effective anti-angiogenesis in prevention or treatment of liver cirrhosis. It is gratifying that celecoxib in combination with octreotide not only arrested intrahepatic and extrahepatic angiogenesis but also reduced the formation of micro-hAPF synergistically. As a result, liver fibrosis was impressively ameliorated and the portal venous pressure decreased by 34 %.

VEGF is a pivotal regulatory protein in angiogenesis. The up-regulation of VEGF, VEGF receptor-2 and PDGF-BB has been verified in animal models of portal hypertension and liver fibrosis [19–21]. The antagonists of VEGF receptor 2, such as Sorafenib, Sunitinib, and combination of Imatinib and Rapamycin may decrease portal hypertension and ameliorate liver fibrosis through the anti-angiogenesis [20, 22, 23]. Similar to Sorafenib, Sunitinib and Imatinib, which inhibit angiogenesis via blockade of VEGF signaling pathway by decreasing VEGF receptor-2, or VEGF [20, 22–24], inhibition of angiogenesis in liver and intestine was also observed when celecoxib was combined with octreotide to suppress VEGF in this study. Anti-angiogenesis strategy was wildly used in the metronomic chemotherapy of solid cancer. A few angiogenesis inhibitors have been tested or approved in the treatment of HCC [25], but they are not recommended for patients with liver cirrhosis partly because of their toxicities and side effects [26, 27].

Consistent with previous reports [19–21, 28], the angiogenesis was associated with up-regulated proangiogenic molecule (VEGF, PDGF-BB and FGF-2) in cirrhotic liver induced by TAA in this study. Moreover, it has been reported that HIF-1α/VEGF, endothelial nitric oxide synthase, MAPK-ERK, JNK-p38 and TGF-β1/Smads signaling pathway were involved in effect of celecoxib and octreotide on the anti-angiogenesis [15, 29]. The p-ERK–HIF-1α pathway was up-regulated in the cirrhotic liver of this study. Celecoxib in combination with octreotide led to down-regulation of p-ERK–HIF-1α pathway in vivo. This synergetic inhibitory effect of celecoxib and octreotide on p-ERK–c-Fos–HIF-1α was further verified in vitro. Among the integrated signal pathways regulating VEGF, the MAPK-ERK signaling pathway involves in the crosstalk of COX-2 and SST signal transduction [30, 31]. Blockade of MAPK-ERK signaling pathway, either by a MEK inhibitor AZD6244 or by combination of celecoxib and octreotide, not only led to significant inhibition of p-ERK, HIF-1α, VEGF and angiogenesis in vitro, but also prevented HIF-1α from binding to VEGF promoter.

PGE2, one of catalysates of COX-2, is involved in modulation of angiogenesis and fibrosis via its receptors [32]. The over-expressions of COX-2 and PGE2 in cirrhotic liver were substantially suppressed by celecoxib plus octreotide in vivo. By using PGE2 and EP-2 inhibitor (AH6809), the experiment showed that celecoxib exerted its anti-angiogenesis effect via COX-2–PGE2–EP-2–p-ERK–VEGF pathway. The anti-angiogenetic effects of octreotide are usually mediated by SSTR-2, SSTR-3 and SSTR-5 [15, 30]. However, this study indicated that SSTR-2 was the only factor which octreotide predominantly activated.

Celecoxib has been widely used in the clinical treatment of osteoarthritis and rheumatoid arthritis [33]. Several studies have demonstrated that celecoxib could efficiently ameliorate portal hypertension and fibrosis in several animal models [6, 12, 13, 34]. Octreotide is indicated for the management of bleeding from gastroesophageal varices in patients with cirrhosis [14]. The regime to combine celecoxib with octreotide was firstly investigated in the rat model with cirrhotic portal hypertension in our study. Both drugs were administrated since the beginning of TAA treatment. Therefore, this regime might be beneficial to early stage of cirrhotic portal hypertension.

The main adverse reactions of nonsteroidal anti-inflammatory drugs (NSAIDs) are gastrointestinal and cardiovascular toxicities. However, celecoxib showed very low toxicity in a metronomic chemotherapy of mammary adenocarcinomas and advanced refractory gastrointestinal cancers [8–10, 35]. Moreover, compared with nonselective NSAIDs, celecoxib was associated with a low risk of clinically significant upper and/or lower gastrointestinal adverse events in patients with osteoarthritis or liver cirrhosis [36, 37].

**Fig. 8:**
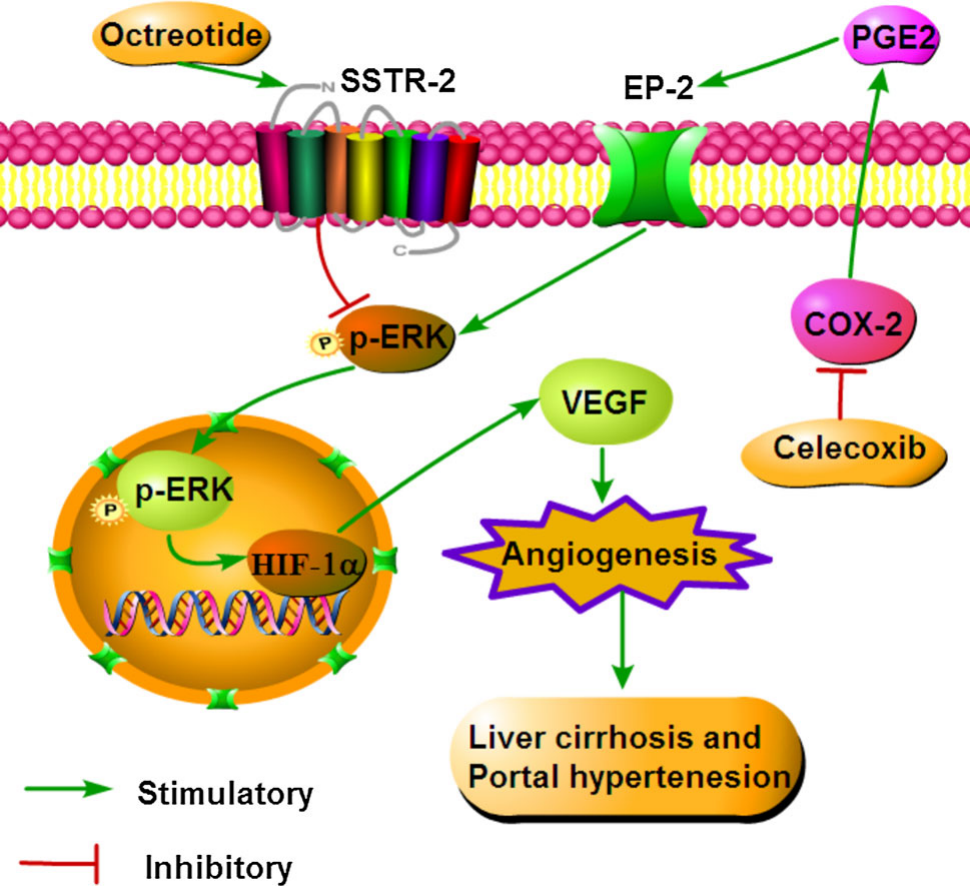
The mechanism schematic model of celecoxib in combination with octreotide in liver cirrhosis and portal hypertension

In conclusion, combination of celecoxib and octreotide synergistically ameliorated liver fibrosis and portal hypertension of the cirrhotic rats induced by TAA via the inhibition of intrahepatic and extrahepatic angiogenesis. The potential mechanisms behind the regimen may due to the inactivation of p-ERK–HIF-1α–VEGF signaling pathway (Fig. 8). The impressive regime may stimulate corresponding trial in the patients with early stage of cirrhotic portal hypertension.

## Electronic supplementary material

Below is the link to the electronic supplementary material.

Supplementary material 1 (DOC 2455 kb)

## Abbreviations

VEGF: Vascular endothelial growth factor
PDGF-BB: Platelet-derived growth factor BB chain
HCC: Hepatocellular carcinoma
COX-2: Cyclooxygenase-2
SST: Somatostatin
SSTRs: Somatostatin receptors
TAA: Thioacetamide
α-SMA: Alpha smooth muscle actin
MT: Masson’s trichrome staining
HE: Hematoxylin and eosin staining
micro-hAPF: Micro-hepatic arterioportal fistulas
HIF-1α: Hypoxia-inducible factor-1α
ERK: Extracellular signal-regulated kinase
p-ERK: Phosphorylated ERK
PGE2: Prostaglandin E2
DMSO: Dimethyl sulfoxide
HUVEC: Human umbilical vein endothelial cells
DMEM: Dulbecco’s modified Eagle’s medium
qRT-PCR: Quantitative real-time PCR
IHC: Immunohistochemistry
NSAIDs: Nonsteroidal anti-inflammatory drugs

## References

[1] Bosch J, Abraldes JG, Fernandez M, Garcia-Pagan JC (2010). Hepatic endothelial dysfunction and abnormal angiogenesis: new targets in the treatment of portal hypertension. J Hepatol.

[2] Bloom S, Kemp W, Lubel J (2015). Portal hypertension: pathophysiology, diagnosis and management. Intern Med J.

[3] Fernandez M (2015). Molecular pathophysiology of portal hypertension. Hepatology.

[4] Thabut D, Shah V (2010). Intrahepatic angiogenesis and sinusoidal remodeling in chronic liver disease: new targets for the treatment of portal hypertension?. J Hepatol.

[5] Blois SM, Piccioni F, Freitag N, Tirado-Gonzalez I, Moschansky P, Lloyd R, Hensel-Wiegel K, Rose M, Garcia MG, Alaniz LD, Mazzolini G (2014). Dendritic cells regulate angiogenesis associated with liver fibrogenesis. Angiogenesis.

[6] Yamamoto H, Kondo M, Nakamori S, Nagano H, Ki Wakasa, Sugita Y, Chang DJ, Kobayashi S, Damdinsuren B, Dono K, Umeshita K, Sekimoto M, Sakon M, Matsuura N, Monden M (2003). JTE-522, a cyclooxygenase-2 inhibitor, is an effective chemopreventive agent against rat experimental liver fibrosis. Gastroenterology.

[7] Sahin M, Sahin E, Gumuslu S (2009). Cyclooxygenase-2 in cancer and angiogenesis. Angiology.

[8] Mainetti LE, Rozados VR, Rossa A, Bonfil RD, Scharovsky OG (2011). Antitumoral and antimetastatic effects of metronomic chemotherapy with cyclophosphamide combined with celecoxib on murine mammary adenocarcinomas. J Cancer Res Clin Oncol.

[9] Perroud HA, Rico MJ, Alasino CM, Queralt F, Mainetti LE, Pezzotto SM, Rozados VR, Scharovsky OG (2013). Safety and therapeutic effect of metronomic chemotherapy with cyclophosphamide and celecoxib in advanced breast cancer patients. Future Oncol.

[10] Allegrini G, Di Desidero T, Barletta MT, Fioravanti A, Orlandi P, Canu B, Chericoni S, Loupakis F, Di Paolo A, Masi G, Fontana A, Lucchesi S, Arrighi G, Giusiani M, Ciarlo A, Brandi G, Danesi R, Kerbel RS, Falcone A, Bocci G (2012). Clinical, pharmacokinetic and pharmacodynamic evaluations of metronomic UFT and cyclophosphamide plus celecoxib in patients with advanced refractory gastrointestinal cancers. Angiogenesis.

[11] Perroud HA, Alasino CM, Rico MJ, Mainetti LE, Queralt F, Pezzotto SM, Rozados VR, Graciela Scharovsky O (2016). Metastatic breast cancer patients treated with low-dose metronomic chemotherapy with cyclophosphamide and celecoxib: clinical outcomes and biomarkers of response. Cancer Chemother Pharmacol.

[12] Wen SL, Gao JH, Yang WJ, Lu YY, Tong H, Huang ZY, Liu ZX, Tang CW (2014). Celecoxib attenuates hepatic cirrhosis through inhibition of epithelial-to-mesenchymal transition of hepatocytes. J Gastroenterol Hepatol.

[13] Gao JH, Wen SL, Yang WJ, Lu YY, Tong H, Huang ZY, Liu ZX, Tang CW (2013). Celecoxib ameliorates portal hypertension of the cirrhotic rats through the dual inhibitory effects on the intrahepatic fibrosis and angiogenesis. PLoS One.

[14] Gracia-Sancho J, Maeso-Diaz R, Bosch J (2015). Pathophysiology and a rational basis of therapy. Dig Dis.

[15] Mejias M, Garcia-Pras E, Tiani C, Bosch J, Fernandez M (2008). The somatostatin analogue octreotide inhibits angiogenesis in the earliest, but not in advanced, stages of portal hypertension in rats. J Cell Mol Med.

[16] Tong H, Li X, Zhang CL, Gao JH, Wen SL, Huang ZY, Wen FQ, Fu P, Tang CW (2013). Transcatheter arterial embolization followed by octreotide and celecoxib synergistically prolongs survival of rabbits with hepatic VX2 allografts. J Dig Dis.

[17] Xie Y, Chen S, Wang CH, Tang CW (2011). SOM230 combined with celecoxib prolongs the survival in nude mice with HepG-2 xenografts. Cancer Biol Ther.

[18] Fernandez M, Vizzutti F, Garcia-Pagan JC, Rodes J, Bosch J (2004). Anti-VEGF receptor-2 monoclonal antibody prevents portal-systemic collateral vessel formation in portal hypertensive mice. Gastroenterology.

[19] Corpechot C, Barbu V, Wendum D, Kinnman N, Rey C, Poupon R, Housset C, Rosmorduc O (2002). Hypoxia-induced VEGF and collagen I expressions are associated with angiogenesis and fibrogenesis in experimental cirrhosis. Hepatology.

[20] Tugues S, Fernandez-Varo G, Munoz-Luque J, Ros J, Arroyo V, Rodes J, Friedman SL, Carmeliet P, Jimenez W, Morales-Ruiz M (2007). Antiangiogenic treatment with sunitinib ameliorates inflammatory infiltrate, fibrosis, and portal pressure in cirrhotic rats. Hepatology.

[21] Van Steenkiste C, Ribera J, Geerts A, Pauta M, Tugues S, Casteleyn C, Libbrecht L, Olievier K, Schroyen B, Reynaert H, van Grunsven LA, Blomme B, Coulon S, Heindryckx F, De Vos M, Stassen JM, Vinckier S, Altamirano J, Bataller R, Carmeliet P, Van Vlierberghe H, Colle I, Morales-Ruiz M (2011). Inhibition of placental growth factor activity reduces the severity of fibrosis, inflammation, and portal hypertension in cirrhotic mice. Hepatology.

[22] Mejias M, Garcia-Pras E, Tiani C, Miquel R, Bosch J, Fernandez M (2009). Beneficial effects of sorafenib on splanchnic, intrahepatic, and portocollateral circulations in portal hypertensive and cirrhotic rats. Hepatology.

[23] Fernandez M, Mejias M, Garcia-Pras E, Mendez R, Garcia-Pagan JC, Bosch J (2007). Reversal of portal hypertension and hyperdynamic splanchnic circulation by combined vascular endothelial growth factor and platelet-derived growth factor blockade in rats. Hepatology.

[24] Thabut D, Routray C, Lomberk G, Shergill U, Glaser K, Huebert R, Patel L, Masyuk T, Blechacz B, Vercnocke A, Ritman E, Ehman R, Urrutia R, Shah V (2011). Complementary vascular and matrix regulatory pathways underlie the beneficial mechanism of action of sorafenib in liver fibrosis. Hepatology.

[25] Sampat KR, O’Neil B (2013). Antiangiogenic therapies for advanced hepatocellular carcinoma. Oncologist.

[26] Alameddine RS, Yakan AS, Skouri H, Mukherji D, Temraz S, Shamseddine A (2015). Cardiac and vascular toxicities of angiogenesis inhibitors: the other side of the coin. Crit Rev Oncol Hematol.

[27] Chen HX, Cleck JN (2009). Adverse effects of anticancer agents that target the VEGF pathway. Nat Rev Clin Oncol.

[28] Pan RL, Xiang LX, Wang P, Liu XY, Nie L, Huang W, Shao JZ (2015). Low-molecular-weight fibroblast growth factor 2 attenuates hepatic fibrosis by epigenetic down-regulation of Delta-like1. Hepatology.

[29] Wang R, Guo L, Wang P, Yang W, Lu Y, Huang Z, Tang C (2013). Chemoprevention of cancers in gastrointestinal tract with cyclooxygenase 2 inhibitors. Curr Pharm Des.

[30] Florio T, Morini M, Villa V, Arena S, Corsaro A, Thellung S, Culler MD, Pfeffer U, Noonan DM, Schettini G, Albini A (2003). Somatostatin inhibits tumor angiogenesis and growth via somatostatin receptor-3-mediated regulation of endothelial nitric oxide synthase and mitogen-activated protein kinase activities. Endocrinology.

[31] Wang YQ, Luk JM, Chu AC, Ikeda K, Man K, Kaneda K, Fan ST (2006). TNP-470 blockage of VEGF synthesis is dependent on MAPK/COX-2 signaling pathway in PDGF-BB-activated hepatic stellate cells. Biochem Biophys Res Commun.

[32] O’Brien AJ, Fullerton JN, Massey KA, Auld G, Sewell G, James S, Newson J, Karra E, Winstanley A, Alazawi W, Garcia-Martinez R, Cordoba J, Nicolaou A, Gilroy DW (2014). Immunosuppression in acutely decompensated cirrhosis is mediated by prostaglandin E2. Nat Med.

[33] McCormack PL (2011). Celecoxib: a review of its use for symptomatic relief in the treatment of osteoarthritis, rheumatoid arthritis and ankylosing spondylitis. Drugs.

[34] Paik YH, Kim JK, Lee JI, Kang SH, Kim DY, An SH, Lee SJ, Lee DK, Han KH, Chon CY, Lee SI, Lee KS, Brenner DA (2009). Celecoxib induces hepatic stellate cell apoptosis through inhibition of Akt activation and suppresses hepatic fibrosis in rats. Gut.

[35] Rico MJ, Perroud HA, Mainetti LE, Rozados VR, Scharovsky OG (2014). Comparative effectiveness of two metronomic chemotherapy schedules-our experience in the preclinical field. Cancer Invest.

[36] Cryer B, Li C, Simon LS, Singh G, Stillman MJ, Berger MF (2013). GI-REASONS: a novel 6-month, prospective, randomized, open-label, blinded endpoint (PROBE) trial. Am J Gastroenterol.

[37] Lee YC, Chang CH, Lin JW, Chen HC, Lin MS, Lai MS (2012). Non-steroidal anti-inflammatory drugs use and risk of upper gastrointestinal adverse events in cirrhotic patients. Liver Int.

