# Correction: Celecoxib and octreotide synergistically ameliorate portal hypertension via inhibition of angiogenesis in cirrhotic rats

**DOI:** 10.1007/s10456-022-09855-4

**Published:** 2022-09-10

**Authors:** Jin-Hang Gao, Shi-Lei Wen, Shi Feng, Wen-Juan Yang, Yao-Yao Lu, Huan Tong, Rui Liu, Shi-Hang Tang, Zhi-Yin Huang, Ying-Mei Tang, Jin-Hui Yang, Hui-Qi Xie, Cheng-Wei Tang

**Affiliations:** 1 Division of Peptides Related With Human Diseases, State Key Laboratory of Biotherapy, West China Hospital, Sichuan University, Chengdu, People’s Republic of China; 2 Department of Gastroenterology, West China Hospital, Sichuan University, Chengdu, 610041 People’s Republic of China; 3 Department of Human Anatomy, Academy of Preclinical and Forensic Medicine, West China Medicine College, Sichuan University, Chengdu, People’s Republic of China; 4 Laboratory of Stem Cell and Tissue Engineering, State Key Laboratory of Biotherapy and Regenerative Medicine Research Center, West China Hospital, Sichuan University, Chengdu, People’s Republic of China; 5 Department of Gastroenterology, The Second Affiliated Hospital of Kunming Medical University, Kunming, People’s Republic of China

## Correction: Angiogenesis (2016) 19:501–511 10.1007/s10456-016-9522-9

Figures 1a, 4c, 5h, and 7f were published incorrectly in the original publication, and the correct figures are provided in this correction. The image of the control group in Fig. 1a and immunohistochemistry of p-ERK in the TAA group were reused from *Gao JH, et al., PLoS One 2013;8:e69309*. In Fig. 1a, the macroscopic view and Masson staining of the control group have been replaced by an image in the same cohort. The image for p-ERK in the TAA group was changed in Fig. 4c by a respective image in the same cohort. In Figs. 5h and 7f, the blot of ERK and p-ERK was replaced by images from the same membrane, respectively. In the supporting information, the anesthetic has been corrected as sodium pentobarbital. At the end of respective treatments, hemodynamic measurements were performed under anesthesia with sodium pentobarbital. The rats were deeply anesthetized with sodium pentobarbital.

**Fig. 1:**
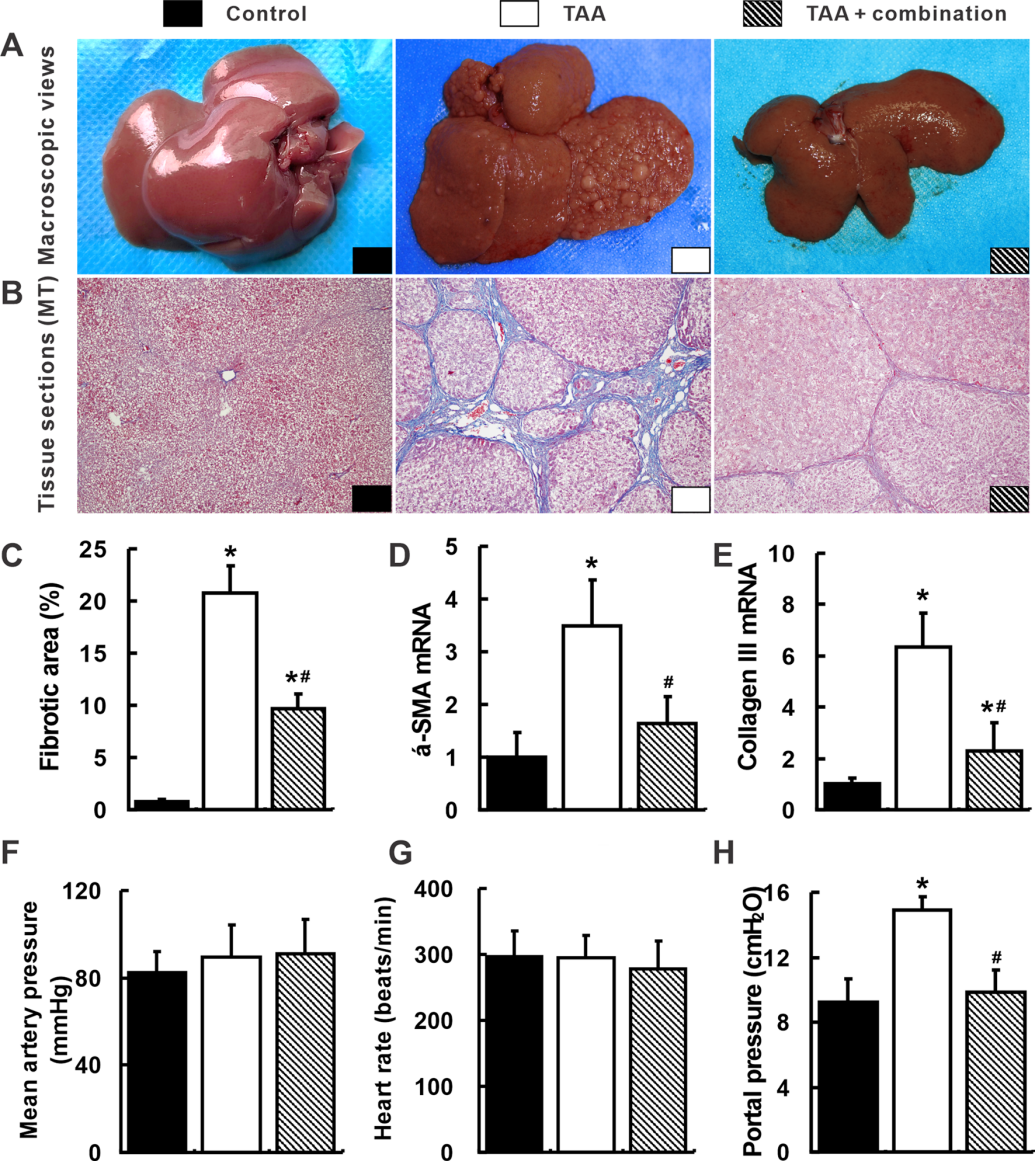
Attenuation of liver cirrhosis and portal hypertension by the combination treatment. Typical cirrhotic appearance with extensive nodular formation (**a**) and fibrotic septa (**b**) was presented in livers of the TAA group. These hepatic nodules and fibrotic septa (Masson’s trichrome staining, × 100 magnifications) were almost not observed in the TAA + combination group. The fibrotic areas (**c**) and hepatic α-SMA (**d**) and collagen III (**e**) mRNA quantified by quantitative real-time PCR (qRT-PCR) also decreased in the TAA + combination group. Mean arterial pressure (**f**) and heart rate (**g**) were comparable in three groups. Portal pressure in the TAA group was the highest among three groups (**h**). *p < 0.05 versus control group; ^#^*p* < 0.05 versus TAA group

**Fig. 4:**
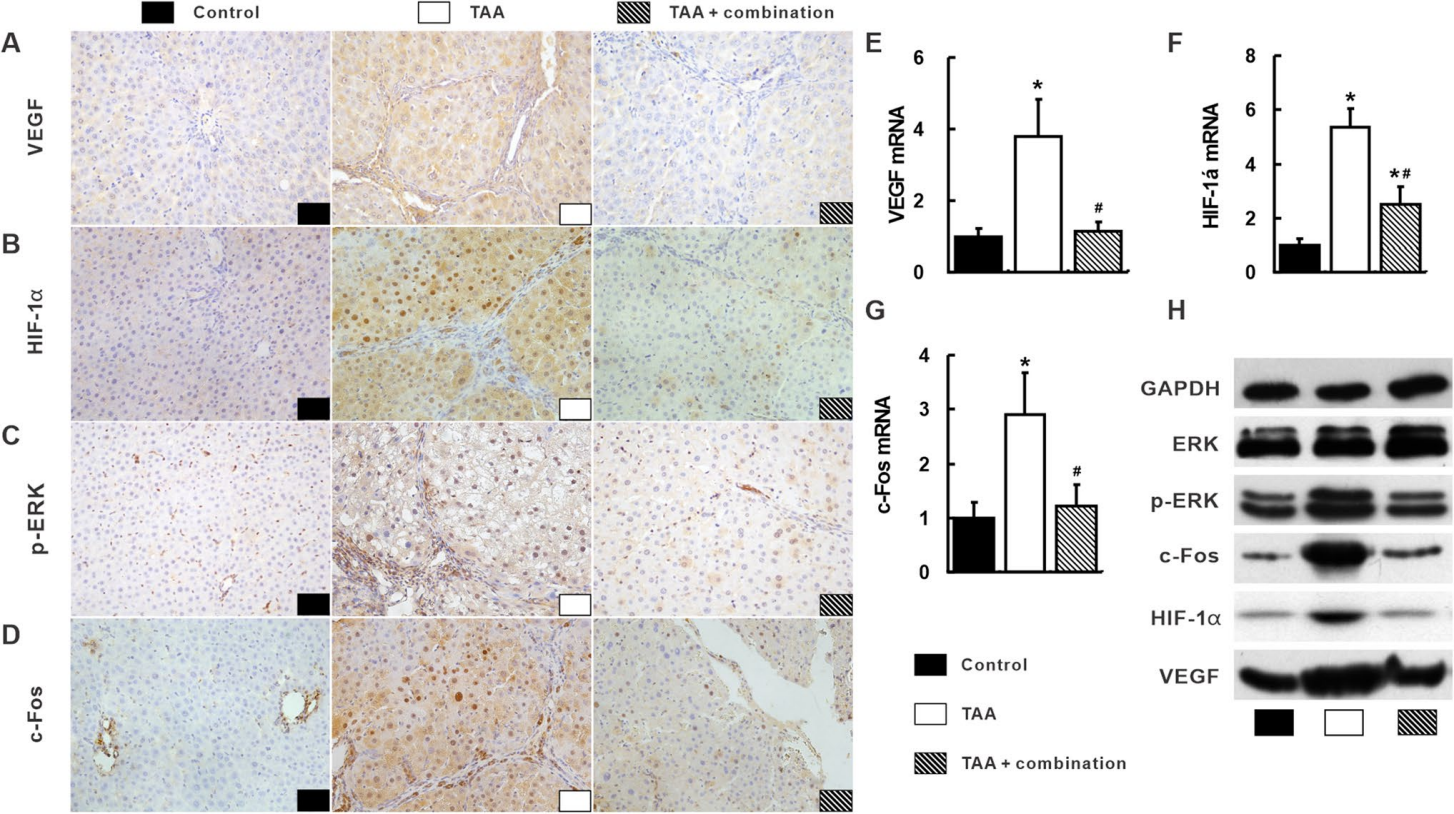
Suppression of the integrated signal pathways with the combination treatment in the liver. Most positive staining of VEGF (**a**), HIF-1α (**b**), p-ERK (**c**) and c-Fos (**d**) visualized by IHC were observed in the TAA group. Consistently, VEGF (**e**), HIF-1α (**f**) and c-Fos (**g**) mRNA and protein (**h**) and p-ERK protein (**h**) in the TAA group were the highest among three groups. *p < 0.05 versus control group; ^#^*p* < 0.05 versus TAA group

**Fig. 5:**
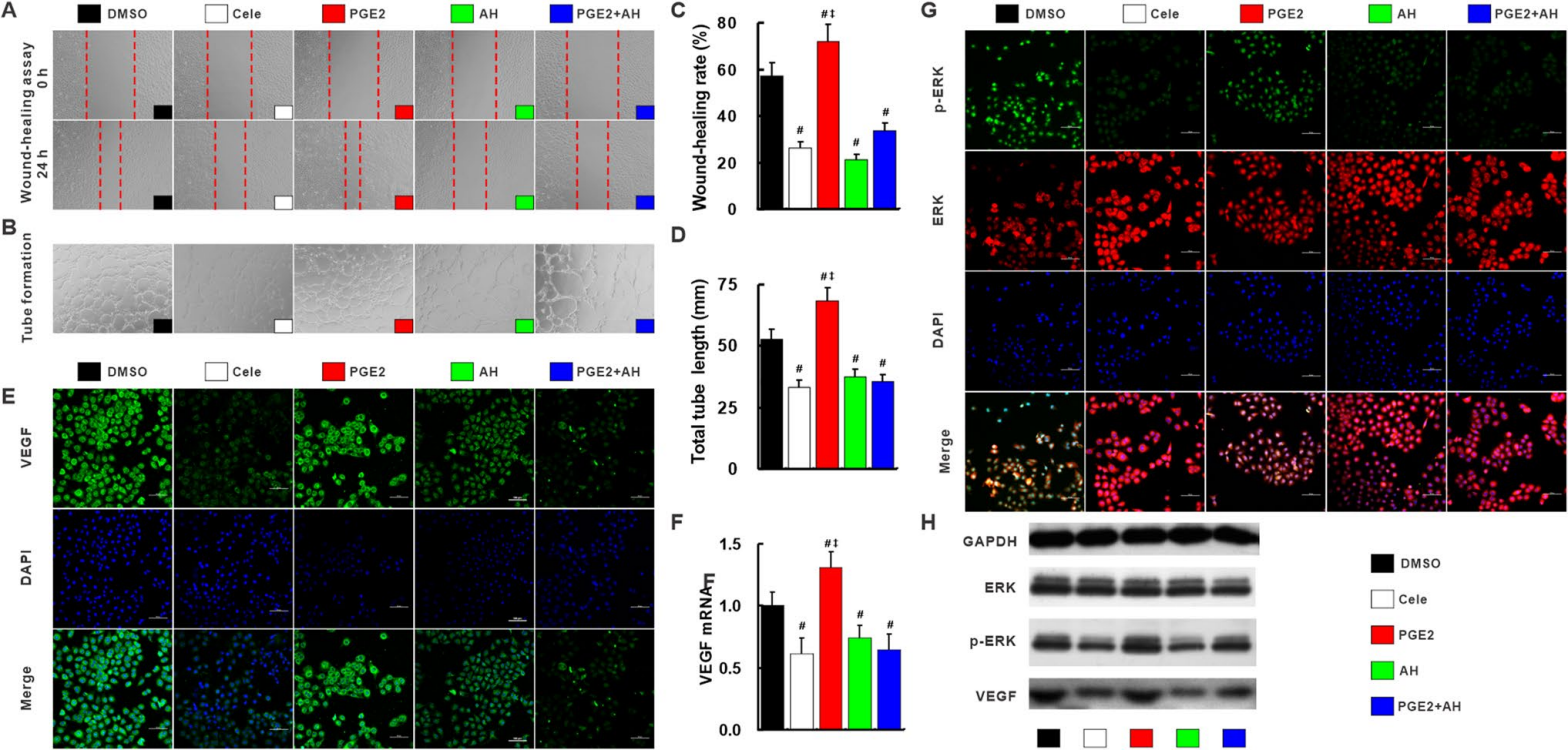
Celecoxib inhibited angiogenesis via inactivation of COX-2/PGE2/EP-2–p-ERK–VEGF signal pathway. Compared with DMSO-treated HUVEC cells, migration rate (**a**, **c**) and tube formation (**b**, **d**) were suppressed by cele, AH and PGE2 + AH, but exacerbated by PGE2 (**a**, **c**). VEGF protein (**e**, **h**) and mRNA (**f**) and p-ERK (**g**, **h**) expression measured by immunofluorescence, and qRT-PCR and Western blot were restored by treatment with cele, AH and PGE2 + AH, but enhanced by treatment with PGE2 compared with DMSO-treated cells. AH, PGE2 receptor EP-2 inhibitor AH6809; Cele, COX-2 inhibitor celecoxib. ^#^*p* < 0.05 versus DMSO; ^‡^*p* < 0.05 versus Cele

**Fig. 7:**
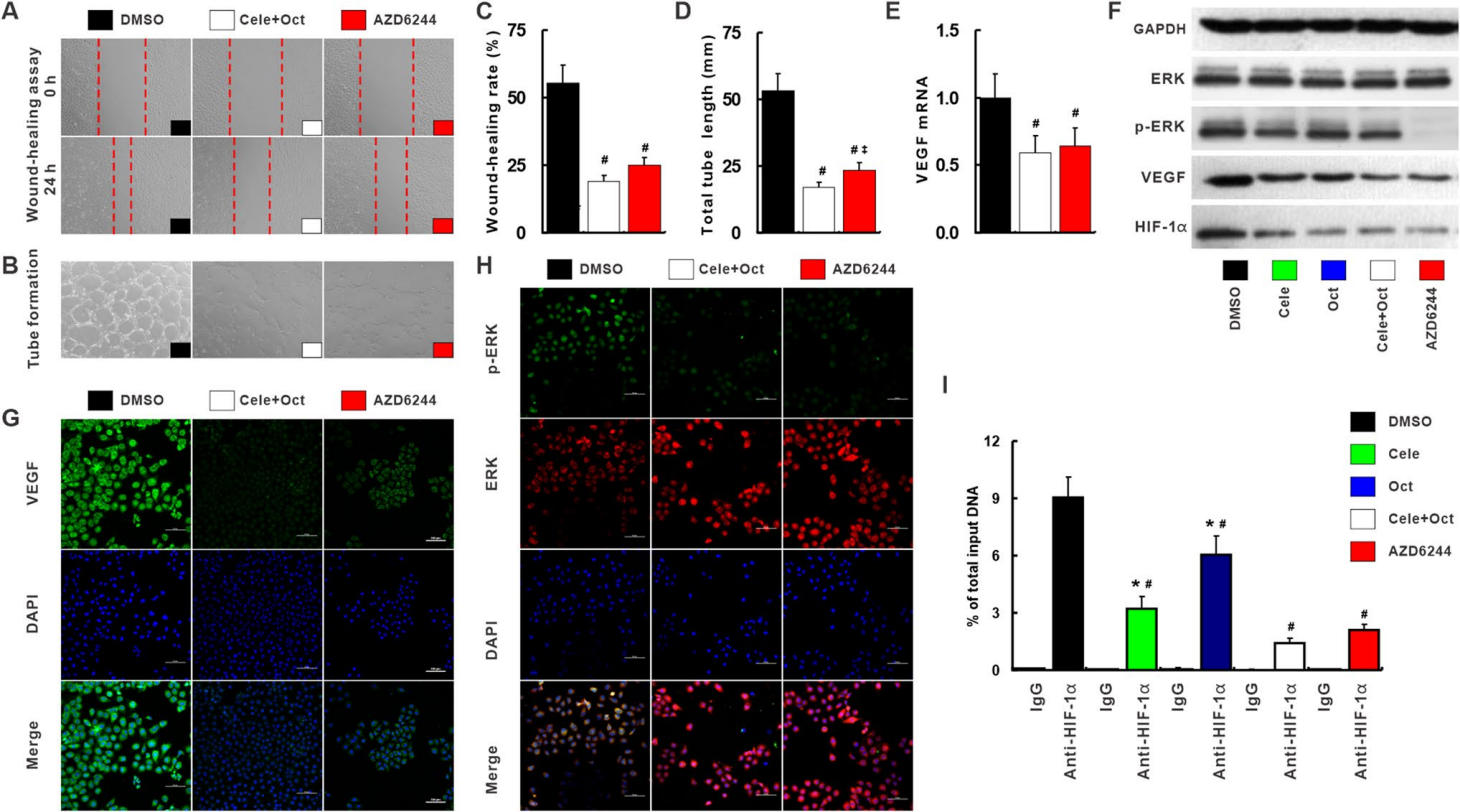
Celecoxib and octreotide synergistically inhibited angiogenesis via p-ERK–HIF-1α–VEGF. Cele + Oct or AZD could significantly reduce the migration rate (**a**, **c**) and tube formation (**b**, **d**) compared with DMSO-treated HUVEC cells. The VEGF mRNA (**e**) and protein (**f**, **g**) p-ERK (**f**, **h**), HIF-1α protein (**h**) determined by immunofluorescence, and qRT-PCR and Western blot were also abolished by Cele, Oct, Cele + Oct or AZD. ChIP assay was to determine HIF-1α binding to VEGF promoter region (**i**). AZD, MEK inhibitor AZD6244. ^#^*p* < 0.05 versus DMSO. *p < 0.05 versus Cele + Oct

