# Supplementary material for: Celecoxib and octreotide synergistically ameliorate portal hypertension via inhibition of angiogenesis in cirrhotic rats
Source: Angiogenesis. 2016 Jul 5;19(4):501–11. doi: 10.1007/s10456-016-9522-9 (PMC5026725; doi:10.1007/s10456-016-9522-9)
Supplement: Supplementary file 1 — Supplementary material 1 (DOC 2455 kb) [file 10456_2016_9522_MOESM1_ESM.doc]

**Angiogenesis**

**Celecoxib and octreotide synergistically ameliorate portal hypertension via inhibition of angiogenesis in cirrhotic rats**

Jin-Hang Gao^#1, 2^, Shi-Lei Wen^#1, 3^, Shi Feng^3^, Wen-Juan Yang^2^, Yao-Yao Lu^2^, Huan Tong^2^, Rui, Liu^1^, Shi-Hang Tang^2^, Zhi-Yin Huang^2^, Ying-Mei Tang^5^, Jin-Hui Yang^5^, Hui-Qi Xie^4^, Cheng-Wei Tang*^1, 2^

*1, Division of Peptides Related with Human Diseases, State Key Laboratory of Biotherapy, West China Hospital, Sichuan University, Chengdu, China.*

*2, Department of Gastroenterology, West China Hospital, Sichuan University, Chengdu, China.*

*3, Department of Human Anatomy, Academy of Preclinical and Forensic Medicine, West China Medicine College, Sichuan University, Chengdu, Sichuan, China.*

*4, Laboratory of Stem Cell and Tissue Engineering, State Key Laboratory of Biotherapy and Regenerative Medicine Research Center, West China Hospital, Sichuan University*

*5, Department of Gastroenterology, The Second Affiliated Hospital of Kunming Medical University, Kunming, Yunnan Province, China.*

**Correspondence*:

Prof. Cheng-Wei Tang,

E-mail: [shcqcdmed@163.com](mailto:shcqcdmed@163.com);

Tel: +86 28 85422383;

Fax: +86 28 85582944

^^Jin-Hang Gao and Shi-Lei Wen contributed equally to this study.

**Details of Methods**

**Hemodynamic measurements of portal pressure**

At the end of respective treatments, hemodynamic measurements were performed under chloral hydrate anesthesia. PE-50 catheters were introduced into the carotid artery and portal vein separately to measure mean arterial pressure, heart rate and portal pressure. The measurements were recorded on a multichannel computer-based recorder BL420 (Taimeng Technology Co., Chengdu, China).

**Histopathological evaluation of cirrhosis and angiogenesis**

Tissues (liver, jejunum, stomach) fixed in 4% paraformaldehyde were embedded in paraffin, sectioned (thickness of 6 μm), and then stained with hematoxylin and eosin (HE) and/or Masson’s trichrome (MT). The morphological changes were examined under a microscope (CX41, Olympus, Tokyo, Japan) equipped with a DP72 digital camera and analyzed by Image-Pro Plus 6.0 software (Media Cybernetics, Silver Spring, MD, USA). The vascular and fibrotic areas in terms of total tissue percentage were assessed by two pathologists in a blinded manner. The vascular areas were accessed based on HE staining sections at ×100 magnifications. The percentages of fibrotic areas were calculated for each MT-stained section at ×100 magnifications. To avoid the sampling bias in the semi-quantitative analysis, a total of 12 pieces of liver tissues from each animal were obtained (3 pieces from each of the left lateral, right medial and right lateral lobes; 1 piece from each of the other 3 small lobes), moreover, three pieces of jejunum and stomach were also collected in each rats.

**Vascular casting of liver vasculature**

Liver vascular corrosion casts were performed in three rats from each group. The rats were deeply anesthetized with chloral hydrate. The livers were first perfused with heparin saline solution (contains 500 U/mL of heparin) through a cannula placed in the portal vein. After that, the vasculature of the rat livers was perfused *in situ* via the portal vein with a freshly prepared solution of methyl methacrylate (Sigma-Aldrich) containing 0.2 g of benzoyl peroxide (Sigma-Aldrich) per 100 mL of resin at a perfusion pressure of 20-30 mmHg. After perfusion, the livers were excised and placed in a water bath for 72 hours to complete the polymerization of the resin, and then the livers were immersed in 15% potassium hydroxide to digest the tissue off the cast. The vascular casts were washed in running water and air-dried. Finally, liver vascular corrosion casts were coated with gold and examined by scanning electronic microscope (SEM).

**Quantitative real-time PCR (qRT-PCR) for mRNA expression**

RNA was extracted from frozen tissue by using TRIzol reagent (Invitrogen). 5 μg of RNA was reverse-transcribed by using a first-strand cDNA kit with random hexamers (Fermentas, Burlington, Canada) and qRT-PCR performed by using the SsoFast EvaGreen PCR master mix (Bio-Rad, Hercules, CA, USA). Intron-spanning primers are listed in supporting table S1. All reactions were run in duplicate by using a CFX96 real-time PCR detection system (Bio-Rad). The mRNA expression was normalized to glyceraldehyde-3-phosphate dehydrogenase (GAPDH) by using the 2^−ΔΔCt^ method and shown as fold changes to the control group.

**Immunohistochemistry staining**

Tissues were processed for paraffin embedding and 3 μm of sections. Sections were deparaffinized in xylene and serial ethanol dilutions. Antigen retrieval was performed by heating sections in 10 mM sodium citrate buffer. Sections were blocked and incubated with primary antibody overnight at 4°C and developed with biotinylated secondary antibodies and then incubated with streptavidin-biotin-complex. The sections were stained with a solution of 3, 3-diaminobenzidine tetrahydrochloride and counterstained with haematoxylin. The primary antibodies used in this study are shown in supporting table S2.

**Western blot analysis for protein expression**

Whole proteins were extracted by using a protein extraction kit (Nanjing Kaiji, Nanjing, China). Equal amounts of proteins (50 μg) from each sample were separated by 10% or 12% SDS-PAGE, transferred to PVDF membrane (Millipore, Billerica, MA, USA). Non-specific binding sites in the membranes were blocked with 5% non-fat dry milk in incubation buffer before addition of primary antibodies (Supporting table S2). Blots were washed and incubated for one hour at room temperature with appropriate HRP-conjugated secondary antibodies. Protein bands were visualized by using an ECL detection kit (BioRad). The autoradiographs were scanned after exposing the membranes to Kodak XAR film (Eastman Kodak, Rochester, NY, USA). Protein expression was determined by using Quantity One software 4.5.0 (Bio-Rad). Protein expressions were normalized to GAPDH.

**Enzyme-linked immunosorbent assay (ELISA) for serum prostaglandin E2 (PGE2)**

The concentration of PGE2 was quantified with an ELISA kit according to the manufacturer’s instructions (USCN Life Sciences Inc, Wuhan, China). Plates were read using the Thermo microplate reader (Thermo Fisher scientific, San Jose, CA, USA) under the wavelengths of 450 nm.

**Ink-gelatin-dextran perfusion for hepatic arterioportal fistulas (hAPF)**

Ink-gelatin-dextran perfusion was performed in five rats from each group. The rats were deeply anesthetized with chloral hydrate. The livers were first heparinized and fixed with 2.5% glutaraldehyde through a cannula placed in the portal vein. Then the ink-gelatin-dextran mixture (5% gelatin and 5% dextran-40 in black ink) were perfused in situ via the portal vein at a perfusion pressure of 30~40 mmHg. After perfusion, a total of 12 pieces of liver tissues from each animal were excised and fixed in 4% paraformaldehyde. The livers were embedded in paraffin, sectioned (thickness of 10 μm, with 4 tissues on each section), and then stained with HE. 3 sections were obtained from each sample with an interval of 3 mm in each section. The hAPF was characterized as ink in the hepatic artery of all 4 tissues on a section. The number of hAPF in each liver was assessed by two pathologists who were blinded to grouping, and displayed as the summation of hAPF in all 36 sections.

**Cell culture and treatments**

Human umbilical vein endothelial cell line (HUVEC) was obtained from the American Type Culture Collection. HUVEC were cultured in Dulbecco's modified Eagle's medium (DMEM, HyClone, Logan, UT, USA) containing 10% fetal bovine serum (FBS, Gibco, Logan, UT, USA) and 100 U penicillin and streptomycin (HyClone). Cells were cultured at 37°C in a humidified atmosphere containing 5% CO_2_. HUVEC cells were in 0.5% FBS DMEM for 24 hours before drug treatment. All treatments in the corresponding concentration were performed in serum-free DMEM for additional 24 hours. Octreotide (0.1 μmol/L), SSTR-2 antagonist (CYN154806, 1 μmol/L) and SSTR-5 antagonist (BIM23056, 1 μmol/L) were dissolved in DMEM. Celecoxib (10 μmol/L), PGE2 (1 μmol/L), PGE2 receptors 2 (EP-2) inhibitor (AH6809, 10 μmol/L) and a highly selective mitogen-activated protein kinase kinase (MEK) inhibitor (AZD6244, 1 μmol/L) were dissolved in dimethyl sulfide (DMSO). PGE2 and corresponding inhibitors were purchased from Sigma-Aldrich.

**Immunocytofluorescence staining for VEGF, ERK and p-ERK**

HUVEC cells were cultured on coverslips at the bottom of 24-well plates. The cells were fixed with 4% paraformaldehyde before permeabilization with 0.5% Triton X-100. After blocking with 1% goat serum, cells were incubated with primary antibodies overnight at 4°C followed by incubation with FITC-conjugated and TRITC-conjugated secondary antibodies. Cell nuclei were stained with 4′, 6-diamino-2-phenylindole (DAPI, Roche, Basel, Switzerland). Slides were mounted with anti-fading medium and were visualized with a confocal microscope (Nikon, Tokyo, Japan).

**In vitro wound healing and cell migration assay**

HUVEC cells were seeded at a density of 5× 10^4^ cells/well in 6-well plates and allowed to form a confluent monolayer. Wound was created by scraping confluent cell monolayer with a 200 μL micropipette tip to denude a strip of monolayer ~100 μm width. Cells were then washed triple with phosphate buffered saline and incubated with corresponding treatments for 24 hours. The images were taken under the inverted microscope at 0 h and 24 h after scratching and the average gap of the wound was calculated by using Image-Pro Plus 6.0 software. The motility was calculated by the formula: (width _0 h_-width _24 h_)/ width _0 h_×100%.

**Tube formation assay and angiogenesis in vitro**

HUVEC cells were seeded at a density of 5× 10^3^ cells/well in Matrigel (BD Bioscience, Billerica, MA, USA) coated 24-well plates in the presence of corresponding treatments. After culture for 24 hours, five different fields were chosen randomly in each well, and photographs were taken under the inverted microscope. The total length per microscopic was measured by using Image-Pro plus 6.0 software.

**Chromatin immunoprecipitation assay (ChIP)**

HUVEC cells were fixed and immunoprecipitated by using EZ Magna ChIP G kit (17-409, Millipore) and Enzymatic ChIP Kit (17-375, Millipore) according to manufacturer’s instructions. HUVEC cells were treated with DMSO, octreotide (0.1 μmol/L), celecoxib (10 μmol/L), octreotide (0.1 μmol/L) + celecoxib (10 μmol/L), AZD6244 (1 μmol/L) for 24 hours, respectively. Cells were fixed with 1% formaldehyde and quenched by 2 mol/L glycine. Cells were collected by scarping in lysis buffer containing protease inhibitor cocktail II. After centrifugation, the cellular pellets were collected. The pellets were re-suspended in EZ-Zyme™ lysis buffer, followed by centrifugation at 2500 g for 10 minutes at 4°C to collect pellet nuclei. Then, pre-warmed EZ-Zyme™ Enzymatic Cocktail was added to cleave DNA into 180~360 nt pieces. The chromatin solutions were precleared and 5 μL of the supernatant was removed as input. The protein-DNA complexes were incubated with mouse anti-HIF-1α (Abcam) or IgG (negative control, Milipore) and rotated overnight at 4°C. Immunocomplexes were affinity absorbed with protein G magnetic beads and collected by centrifugation. Crosslinks were reversed by adding proteinase K contained ChIP elution buffer. Free DNA was purification by using spin columns. The eluates were employed as a template for qPCR with a CFX96 real-time PCR detection system (Bio-Rad). The specific primer for amplifications of VEGF promoter region from -1041 to -835 was revised according to previous publication (supporting table 1).

**Supporting table 1. List of primers for qRT-PCR** and ChIP

| **Gene** | **GenBank Accession No.** |  | **Sequence Primers (5’-3’)** | **Product Size** |
| --- | --- | --- | --- | --- |
| ***CD31-rat*** | [NM_031591.1](http://www.ncbi.nlm.nih.gov/nucleotide/75832032?report=genbank&log$=nucltop&blast_rank=13&RID=4M1DRC7Z015) | F | CTTCACCATCCAGAAGGAAGAGAC | 360 bp |
|  |  | R | CACTGGTATTCCATGTCTCTGGTG |  |
| ***Collagen III-rat*** | [NM_032085.1](http://www.ncbi.nlm.nih.gov/nucleotide/56711253?report=genbank&log$=nucltop&blast_rank=13&RID=4M1DBJCM014) | F | GATGGCTGCACTAAACACACT | 241 bp |
|  |  | R | CACTTTCACTGGTTGACGAGA |  |
| **COX-2-rat** | [NM_017232.3](http://www.ncbi.nlm.nih.gov/nucleotide/148747269?report=genbank&log$=nucltop&blast_rank=4&RID=4M1CUTDC015) | F | GCTCATACTGATAGGAGAGACGA | 117 bp |
|  |  | R | TGGAACTGCTGGTTGAAAAG |  |
| ***ERK-rat*** | [NM_053842.1](http://www.ncbi.nlm.nih.gov/nucleotide/16758697?report=genbank&log$=nucltop&blast_rank=24&RID=4M1PH9BT015) | F | AATAAGGTGCCGTGGAACA | 158 bp |
|  |  | R | GGCTCATCACTTGGGTCAT |  |
| ***c-Fos-rat*** | [NM_022197.2](http://www.ncbi.nlm.nih.gov/nucleotide/148298807?report=genbank&log$=nucltop&blast_rank=7&RID=4M1CEH5A015) | F | AACACACAGGACTTTTGCG | 142 bp |
|  |  | R | CTCTGGTCTGCGATGGG |  |
| ***FGF-2-rat*** | [NM_019305.2](http://www.ncbi.nlm.nih.gov/nucleotide/159032538?report=genbank&log$=nucltop&blast_rank=2&RID=4HPARV4X01N) | F | ACACGTCAAACTACAGCTCCA | 153 bp |
|  |  | R | GACTCCAGGCGTTCAAAGA |  |
| ***GAPDH-rat*** | [NM_017008.4](http://www.ncbi.nlm.nih.gov/nucleotide/402691727?report=genbank&log$=nucltop&blast_rank=4&RID=4M1SGJR4014) | F | TCGGTGTGAACGGATTTG | 173 bp |
|  |  | R | CTCAGCCTTGACTGTGCC |  |
| ***HIF-1α-rat*** | [NM_024359.1](http://www.ncbi.nlm.nih.gov/nucleotide/13242248?report=genbank&log$=nucltop&blast_rank=81&RID=4M1BMPPX014) | F | TTCGGCAGCGATGACAC | 130 bp |
|  |  | R | CAGAGGCAGGTAATGGAGAC |  |
| ***c-Myc-rat*** | [NM_012603.2](http://www.ncbi.nlm.nih.gov/nucleotide/51491887?report=genbank&log$=nucltop&blast_rank=1&RID=4HPCHH5701N) | F | GTCTTCCCCTACCCGCT | 189 bp |
|  |  | R | ATCTTGTTCTTCTTCAGAGTCG |  |
| ***PDGF-B-rat*** | [NM_031524.1](http://www.ncbi.nlm.nih.gov/nucleotide/158081746?report=genbank&log$=nucltop&blast_rank=21&RID=4HPCUUTD01N) | F | ACTCCATCCGCTCCTTTGA | 185 bp |
|  |  | R | GCACTCGGCGATTACGG |  |
| ***α-SMA-rat*** | [NM_031004.2](http://www.ncbi.nlm.nih.gov/nucleotide/148298812?report=genbank&log$=nucltop&blast_rank=62&RID=4M1B1EGA014) | F | CCGAGATCTCACCGACTACC | 120 bp |
|  |  | R | TCCAGAGCGACATAGCACAG |  |
| ***VEGF-rat*** | [NM_031836.3](http://www.ncbi.nlm.nih.gov/nucleotide/560186573?report=genbank&log$=nucltop&blast_rank=76&RID=4M1X3CSF015) | F | CGAACGTACTTGCAGATGTGAC | 139 bp |
|  |  | R | GACGGTGACGATGGTGGT |  |
| ***β-Actin-human*** | [NM_001101.3](http://www.ncbi.nlm.nih.gov/nucleotide/168480144?report=genbank&log$=nucltop&blast_rank=1&RID=4M22TA18014) | F | CACAGAGCCTCGCCTTT | 318 bp |
|  |  | R | GGTGCCAGATTTTCTCCAT |  |
| ***SSTR1-human*** | [NM_001049.2](http://www.ncbi.nlm.nih.gov/nucleotide/33946330?report=genbank&log$=nucltop&blast_rank=1&RID=4HRWRUDN01N) | F | CAACTCTCCAGGCTTAGGG | 189 bp |
|  |  | R | GCGTCAGCAGCCAAAGT |  |
| ***SSTR2-human*** | [NM_001050.2](http://www.ncbi.nlm.nih.gov/nucleotide/44890054?report=genbank&log$=nucltop&blast_rank=1&RID=4HRTBYVN01N) | F | GGCACAAGAGGGTCGAG | 213 bp |
|  |  | R | CCAGCCAGCCCAGAGAT |  |
| ***SSTR3-human*** | [NM_001051.3](http://www.ncbi.nlm.nih.gov/nucleotide/522838248?report=genbank&log$=nucltop&blast_rank=2&RID=4HRW5R9R01N) | F | CCTTTCCCCTCTATTTCTATTG | 373 bp |
|  |  | R | GCTATTTGCCTGCCCAT |  |
| ***SSTR4-human*** | [NM_001052.2](http://www.ncbi.nlm.nih.gov/nucleotide/149944553?report=genbank&log$=nucltop&blast_rank=1&RID=4HRXU86501N) | F | CTTCGCAGACACCAGACC | 277 bp |
|  |  | R | GAAAGGCATCCAGCAGAG |  |
| ***SSTR5-human*** | [NM_001053.3](http://www.ncbi.nlm.nih.gov/nucleotide/289547751?report=genbank&log$=nucltop&blast_rank=2&RID=4HRY3TKA01N) | F | GCTGAGTGGGCACAAATC | 123 bp |
|  |  | R | CATTTATCCTCGCATTTATTC |  |
| ***VEGF-human*** | [NM_003376.5](http://www.ncbi.nlm.nih.gov/nucleotide/284172448?report=genbank&log$=nucltop&blast_rank=18&RID=4M235J8F015) | F | CCATGCCAAGTGGTCCC | 267 bp |
|  |  | R | CGCATAATCTGCATGGTGA |  |
| ***VEGF-ChIP*** | NC_000006.12 | F | CAGGAACAAGGGCCTCTGTCT | 146 bp |
|  |  | R | TGGAGCTGAGAACGGGAAGCT |  |

### F: **Forward primer; R: Reverse primer; COX-2,** cyclooxygenase-2; *ERK,* extracellular signal-regulated kinase; FGF-2, **fibroblast growth factor-2***;* GAPDH, **glyceraldehyde-3-phosphate dehydrogenase;** HIF-1α*,* **Hypoxia-inducible factor 1, alpha subunit;** **PDGF-B,** platelet-derived growth factor subunit B; α-SMA, alpha smooth muscle actin**; SSTR, somatostatin receptor; VEGF,** vascular endothelial growth factor. **VEGF-ChIP, the primer of VGEF for ChIP assay.**

**Supporting table 2. Antibodies list for immunohistochemistry, Immunofluorescence and Western blot**

| Antibodies | Applications | Dilution | Source | Company |
| --- | --- | --- | --- | --- |
| **CD31** | IHC  WB | 1:100  1:200 | Rabbit | Santa Cruz Biotechnology, Santa Cruz, CA, USA |
| **ERK** | IF  WB | 1:200  1:500 | Rabbit | Santa Cruz Biotechnology |
| **p-ERK** | IHC  IF  WB | 1:100  1:100  1:2000 | Mouse | Santa Cruz Biotechnology |
| **c-Fos** | IHC  WB | 1:200  1:500 | Rabbit | Abcam, Cambridge, UK |
| **HIF-1α** | IHC  WB | 1:200  1:500 | Mouse | Abcam |
| **VEGF** | IHC  IF  WB | 1:200  1:300  1:500 | Mouse | Santa Cruz Biotechnology |
| **COX-2** | IHC  WB | 1:100  1:400 | Rabbit | Abcam |
| **GAPDH** | WB | 1:10000 | Mouse | Abcam |

IHC: immunohistochemistry; IF, Immunofluorescence; WB, Western blot; ERK, extracellular signal-regulated kinase; p-ERK, phosphorylated ERK; GAPDH, glyceraldehyde-3-phosphate dehydrogenase; HIF1α, Hypoxia-inducible factor 1, alpha subunit; SSTR, somatostatin receptor; VEGF, vascular endothelial growth factor.


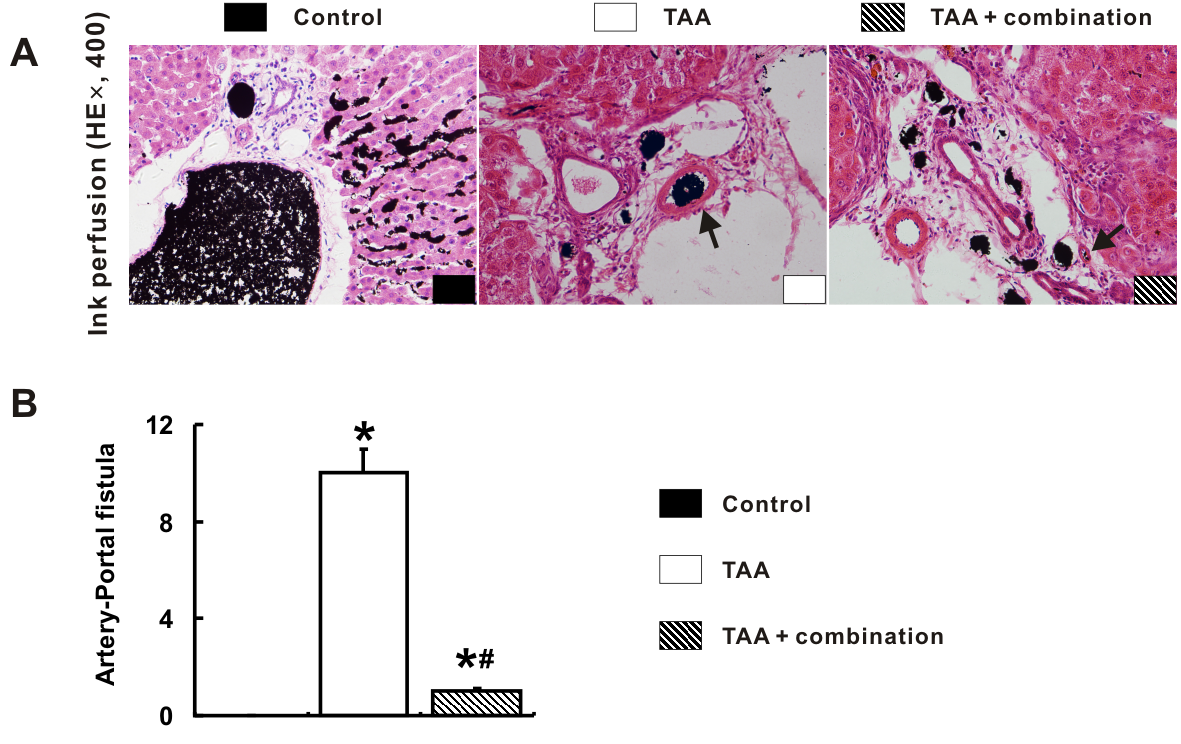


**Supporting Figure S1. Suppression of micro-hAPF by the combination treatment**

Micro-hAPF was not observed in the control group (A). The number of micro-hAPF per liver in the TAA group was the highest (B). Black arrow indicates micro-hAPF. **p*<0.05 *vs*. control group; #*p*<0.05 *vs*. TAA group.

**
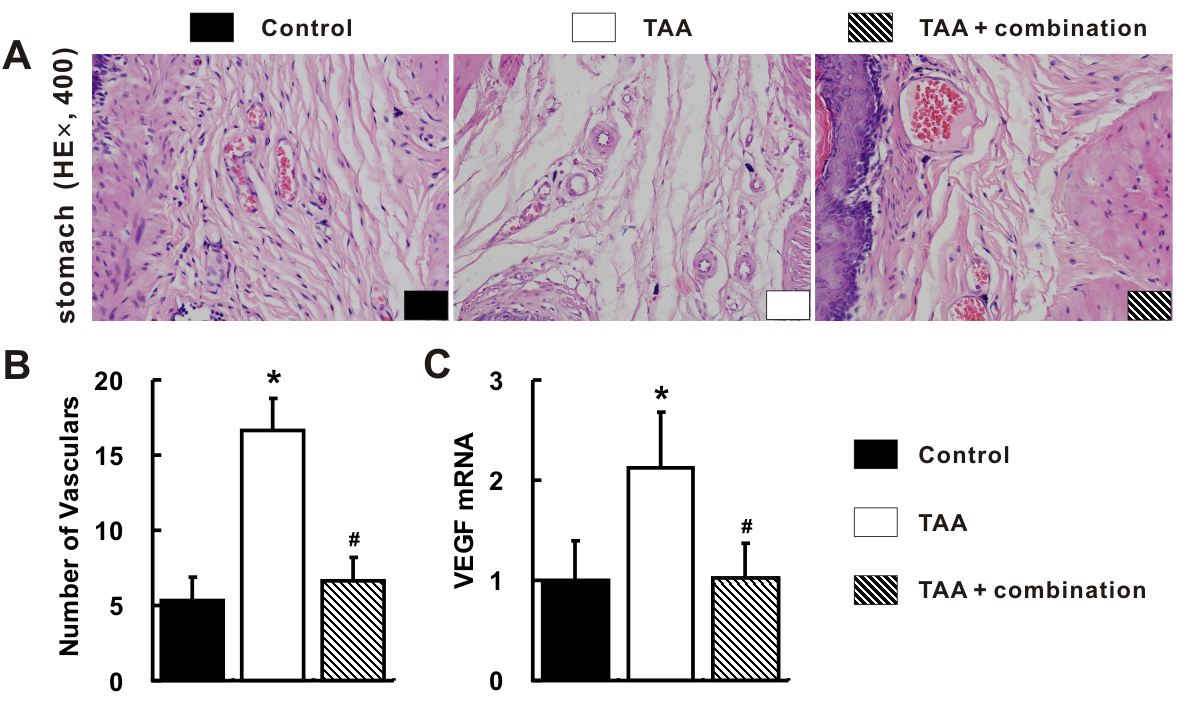
**

**Supporting Figure S2. Inhibition of gastric angiogenesis by the combination treatment**

The enhancement of gastric angiogenesis in the TAA group was visualized by HE (A).

The number of vessels per fields (B) and VEFG mRNA (C) in the TAA group was the highest. **p*<0.05 *vs*. control group; #*p*<0.05 *vs*. TAA group.

**
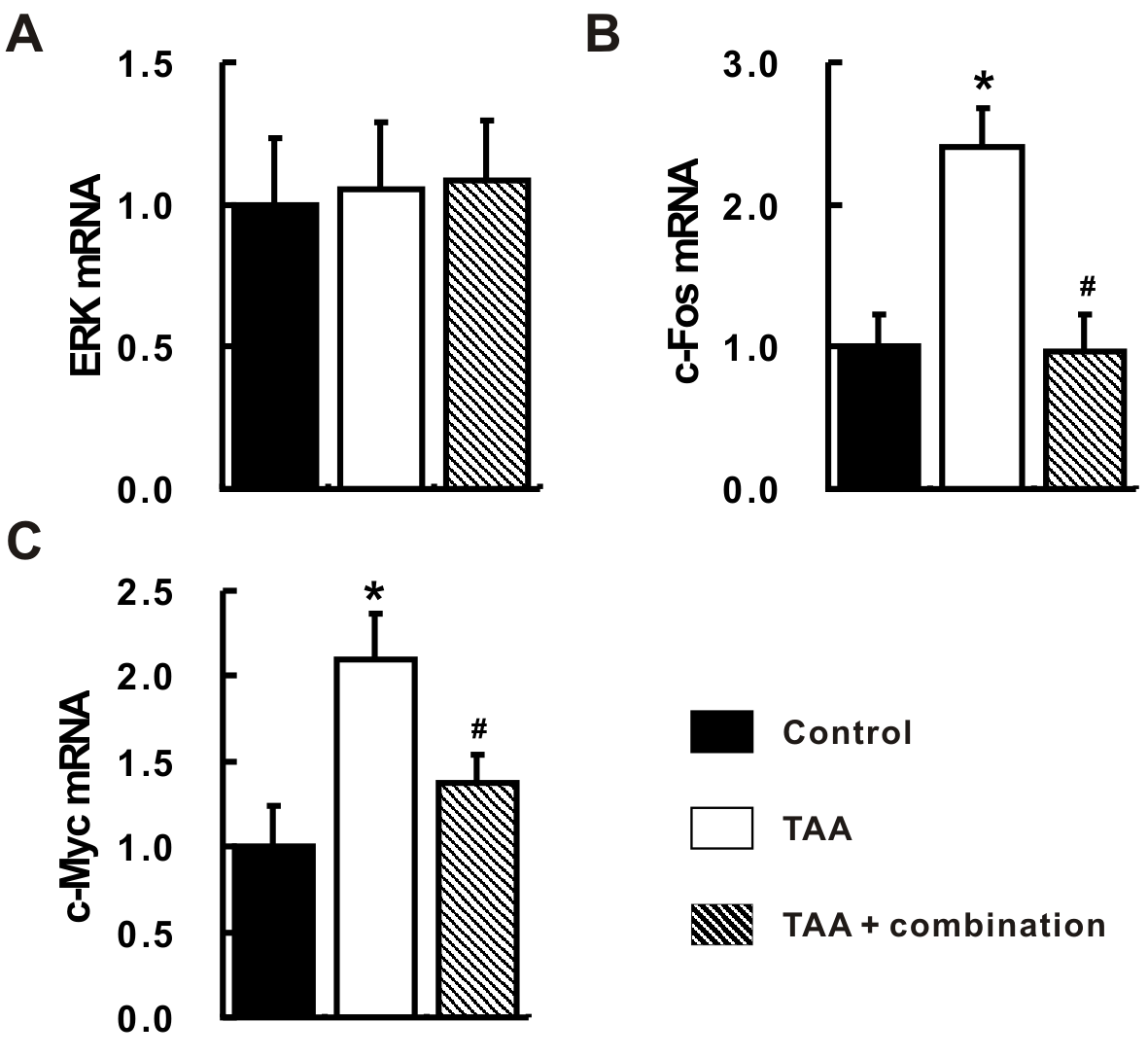
**

**Supporting Figure S3. Effect of the combination treatment on intestinal ERK, c-Fos and c-Myc mRNA expression**

There was no significant difference of intestinal ERK mRNA among three groups (A). Intestinal c-fos mRNA was the highest in the TAA group (B). Intestinal c-Myc mRNA was the highest in the TAA group, while the increase of c-Myc mRNA was inhibited in the TAA + combination group. **p*<0.05 *vs*. control group; #*p*<0.05 *vs*. TAA group.

**
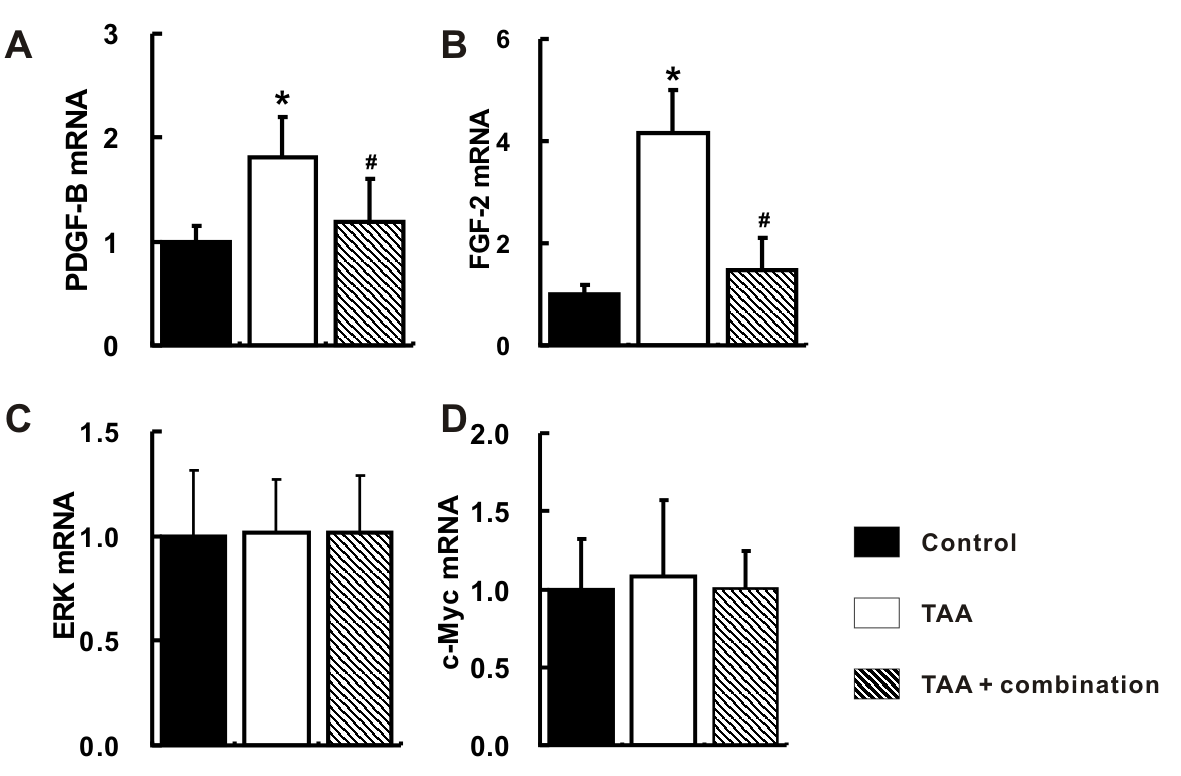
**

**Supporting Figure S4. Reduction of hepatic PDGF-B and FGF-2 mRNA by the combination treatment**

Hepatic PDGF-B and FGF-2 mRNA were the highest in the TAA group (A). There were no significant differences of hepatic ERK and c-Myc mRNA among three groups. **p*<0.05 *vs*. control group; #*p*<0.05 *vs*. TAA group.

**
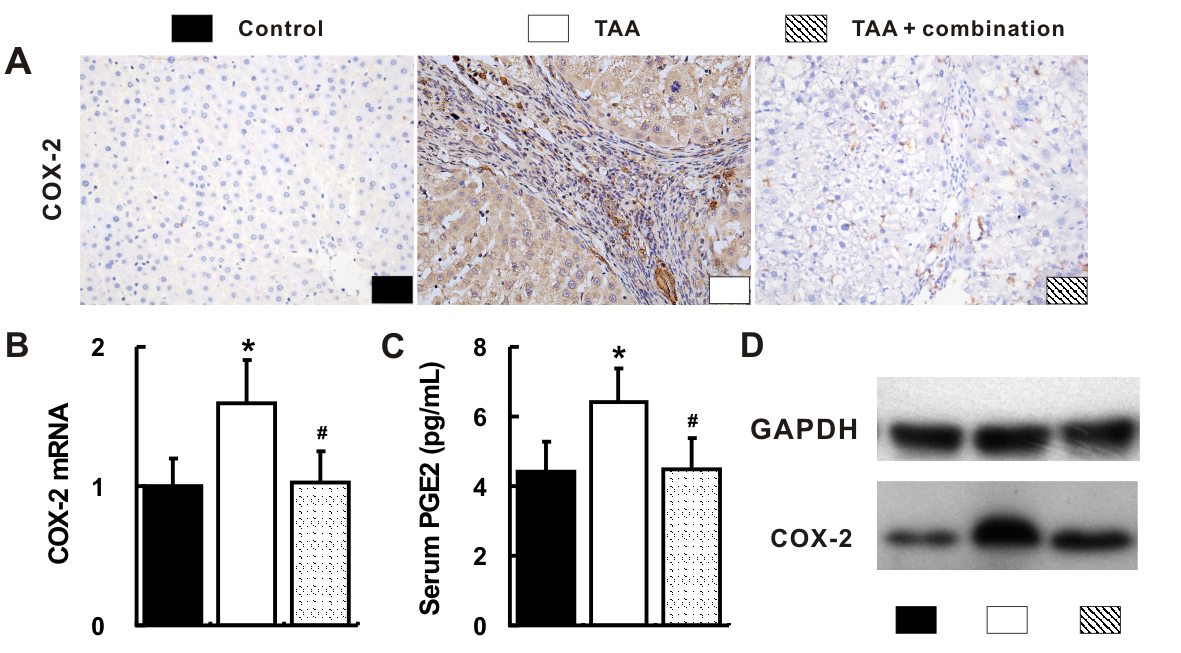
**

**Supporting Figure S5. Suppression of hepatic COX-2 and serum PGE2 by celecoxib**

Comprehensive expression of COX-2 visualize by IHC was revealed in the TAA group (A). The up-regulation of hepatic COX-2 mRNA (B) and protein (D), serum PGE2 (C) induced by TAA were remarkably decreased in the TAA + combination group. **p*<0.05 *vs*. control group; #*p*<0.05 *vs*. TAA group.


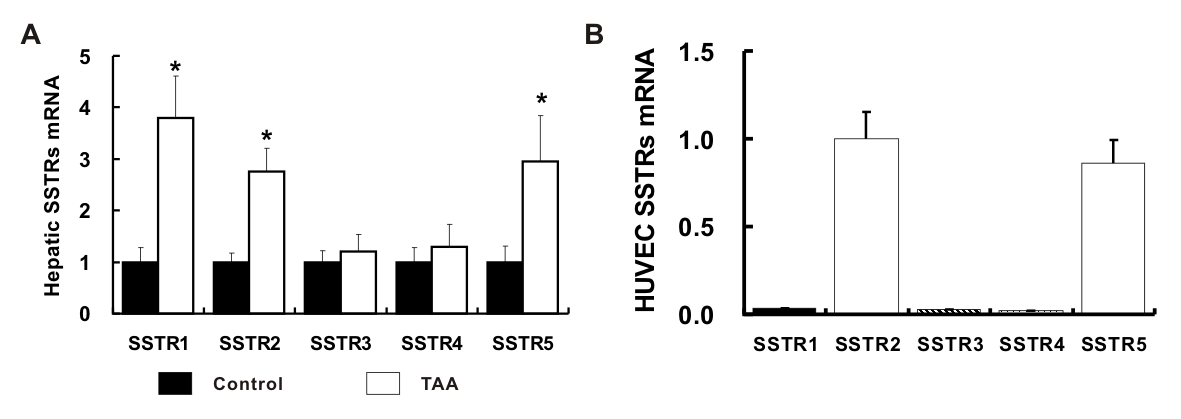


**Supporting Figure S6. Expression of SSTRs in liver and HUVEC cells**

The presence of SSTRs in liver (A) and HUVEC cells (B) was ascertained by qRT-PCR. The mRNA level of SSTR-2 and SSTR-5 was up-regulated in the TAA group compared with that in the control group. Additionally, mRNA of SSTR-2 and SSTR-5 was detectable in HUVEC cells. **p*<0.05 *vs*. control group.
